# Identification and validation of a novel eight mutant-derived long non-coding RNAs signature as a prognostic biomarker for genome instability in low-grade glioma

**DOI:** 10.18632/aging.203079

**Published:** 2021-06-03

**Authors:** Aierpati Maimaiti, Xixian Wang, Yinan Pei, Nuerbiye Nuermaimaiti, Abudireheman Tuersunniyazi, Yaeraili Abula, Zhaohai Feng, Lei Jiang, Xin Shi, Maimaitijiang Kasimu

**Affiliations:** 1 Department of Functional Neurosurgery, Neurosurgery Centre, The First Affiliated Hospital of Xinjiang Medical University, Urumqi, Xinjiang 830054, China; 2 State Key Laboratory of Pathogenesis, Prevention and Treatment of High Incidence Disease in Central Asia, Department of Biochemistry and Molecular Biology, Basic Medicine College, Xinjiang Medical University, Urumqi, Xinjiang 830054, China

**Keywords:** genomic instability-associated lncRNAs signature (GILncSig), long non-coding RNA (lncRNA), low-grade glioma (LGG), risk score, prognosis

## Abstract

Long non-coding RNAs (lncRNAs) comprise an integral part of the eukaryotic transcriptome. Alongside proteins, lncRNAs modulate lncRNA-based gene signatures of unstable transcripts, play a crucial role as antisense lncRNAs to control intracellular homeostasis and are implicated in tumorigenesis. However, the role of genomic instability-associated lncRNAs in low-grade gliomas (LGG) has not been fully explored. In this study, lncRNAs expression and somatic mutation profiles in low-grade glioma genome were used to identify eight novel mutant-derived genomic instability-associated lncRNAs including H19, FLG-AS1, AC091932.1, AC064875.1, AL138767.3, AC010273.2, AC131097.4 and ISX-AS1. Patients from the LGG gene mutagenome atlas were grouped into training and validation sets to test the performance of the signature. The genomic instability-associated lncRNAs signature (GILncSig) was then validated using multiple external cohorts. A total of 59 novel genomic instability-associated lncRNAs in LGG were used for least absolute shrinkage and selection operator (Lasso), single and multifactor Cox regression analysis using the training set. Furthermore, the independent predictive role of risk features in the training and validation sets were evaluated through survival analysis, receiver operating feature analysis and construction of a nomogram. Patients with IDH1 mutation status were grouped into two different risk groups based on the GILncSig score. The low-risk group showed a relatively higher rate of IDH1 mutations compared with patients in the high-risk group. Furthermore, patients in the low-risk group had better prognosis compared with patients in the high-risk group. In summary, this study reports a reliable prognostic prediction signature and provides a basis for further investigation of the role of lncRNAs on genomic instability. In addition, lncRNAs in the signature can be used as new targets for treatment of LGG.

## INTRODUCTION

Low-grade gliomas are a diverse group of heterogeneous brain tumors that originate from glial cells, and their aggressiveness varies depending on subtype and grade. LGG is a grade II-III glioma based on the World Health Organization classification system, and is different from high-grade glioblastoma (GBM) (grade IV glioma) [1, 2]. LGG and GBM exhibit diverse molecular and clinical features [3]. LGG is more prevalent in younger patients unlike other tumors with a mean age of 41 years [4]. LGG accounts for approximately 15% of all brain and CNS tumors, with an incidence of approximately 1 in 100,000 [5]. Currently, the conventional treatment approaches of LGG are surgical treatment and postoperative radiotherapy, however, these methods do not prevent transformation from LGG to GBM or recurrence [6]. Therefore, diagnosis at an early stage of low-grade gliomas can improve clinical prognosis of patients. Further, studies should explore effective biomarkers based on the underlying mechanisms of LGG progression for development of personalized treatment approaches.

Genomic instability changes and malignant proliferation are hallmarks for cancer development [7]. Previous studies report that genomic instability plays a key role in cancer prognosis, and high levels of genomic instability are implicated in survival and progression of tumors [8]. Although the underlying molecular-underpinnings of genomic instability have not been fully explored, abnormalities in transcription and post-transcriptional modulation are linked to genomic instability, indicating that molecular features can be used to quantify genomic instability. For instance, a study by Bao et al. [9] analysed 128 gene expression profiles of breast cancer specimens and identified genomic instability signatures for two genes and lncRNA signatures associated with genomic instability and breast cancer outcomes.

Long noncoding RNAs (lncRNAs) are a class of RNA molecules with transcripts longer than 200 nt that do not encode proteins. They modulate expression levels of transcribed genes at multiple layers including epigenetic modulation, transcriptional modulation and post-transcriptional modulation [10]. Several studies have explored various lncRNAs, their roles in transcriptional interference, post-transcriptional gene silencing (PTGS), genomic imprinting and induction of chromatin remodelling and nucleosome modification, regulation of variable splicing patterns, generation of endogenous siRNAs, differentiation, and regulation of cis and transgene expression. LncRNAs play integral roles in development of various diseases. For instance, dysregulation of lncRNAs expression is implicated in tumor proliferation, tumor progression and metastasis. The lncRNA, Norad which is a recently described noncoding RNA, is a Noncoding RNA activated by DNA damage (Norad) [11]. Previous studies developed lncRNA-Norad-deficient mouse models using CRISPR/Cas9 gene-editing technology [12]. In these models, Norad-deficient mice exhibit a multisystem degenerative phenotype significantly resembling premature aging compared to controls. This phenotype is characterized by PUM overactivity and increased repression of genes essential for normal mitosis, resulting in genomic instability [13]. Notably, NORAD deletion causes severe mitochondrial dysfunction due to upregulation of PUM targets for multiple genes that regulate mitochondrial homeostasis *in vivo* [14]. Previous studies on classification, differentiation and prognosis prediction of gliomas mainly focused on high-grade gliomas or glioblastomas. Therefore, biological markers for prognostic stratification of LGG patients have not been fully explored. The aim of this study was to explore the key role of lncRNAs in maintaining genomic instability in LGG patients.

Genomic stability maintenance is fundamental to all life activities, and DNA damage and replication stress as a result of multiple exogenous and endogenous factors are implicated in genomic instability. A previous study reports that lncRNA NORAD regulates activity of a protein complex composed of RBMX-TOP1 and other proteins that prevent genomic instability by binding to RBMX proteins [15]. In addition, lncRNA NORAD enhances genomic stability by separating PUMILIO proteins from their target mRNAs [16]. Genomic instability and the resulting mutagenicity can cause genetic alterations in cancer cells, promoting tumor progression. Studies report that copy and allelic imbalance of two TP53 mutations in MDS patients results in genomic instability which is significantly associated with lower survival rates [17]. Prognosis of gliomas with IDH1/2 mutations is generally good, however, some gliomas exhibiting IDH1/2 mutations have similar prognosis as glioblastomas without IDH1/2 mutations [18, 19]. Most glioblastomas have a large number of gene copy number and sequence variants at the genomic level. Some glioma samples exhibit highly complex karyotypic, gene copy number and sequence variants in addition to shared copies, leading to heterogeneity between and within glioma samples. Although studies have several lncRNAs involved in genomic stability, lncRNAs associated with genomic instability and the resulting clinical implications in low-grade gliomas have not been fully explored.

In this study, we explored lncRNAs associated with genomic instability in LGG based on lncRNA expression and somatic mutation profiles in the genome of low-grade gliomas. Further, we developed risk models for prognosis prediction and development of novel treatment approaches for LGG patients.

## RESULTS

### Identification of lncRNAs associated to genomic instability in Low-grade glioma patients

**Figure 1:**
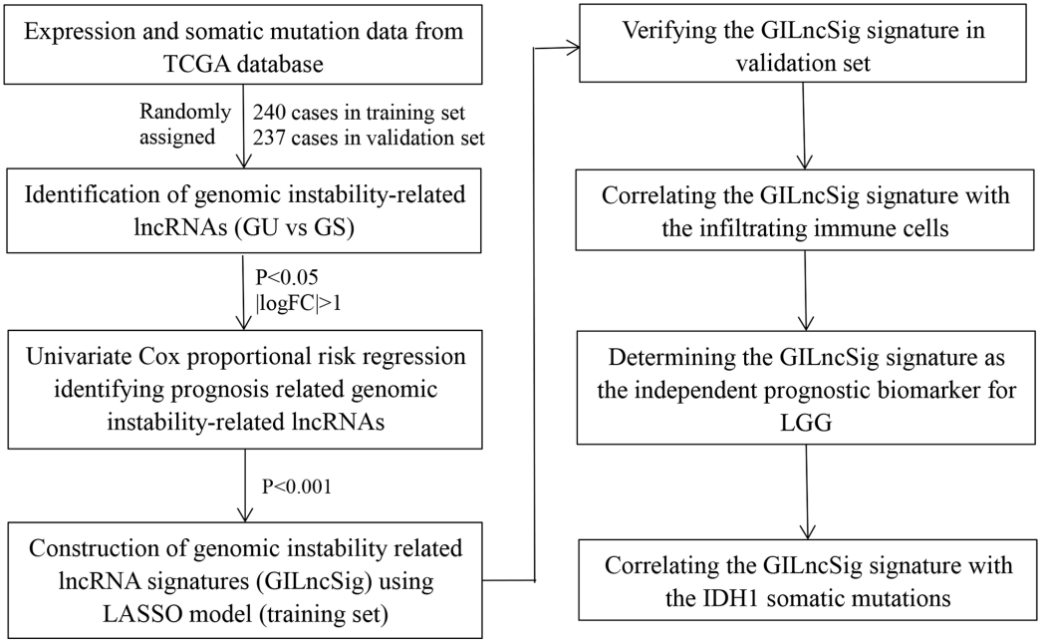
Study flow chart of genomic instability-related lncRNAs construction.

**Figure 2:**
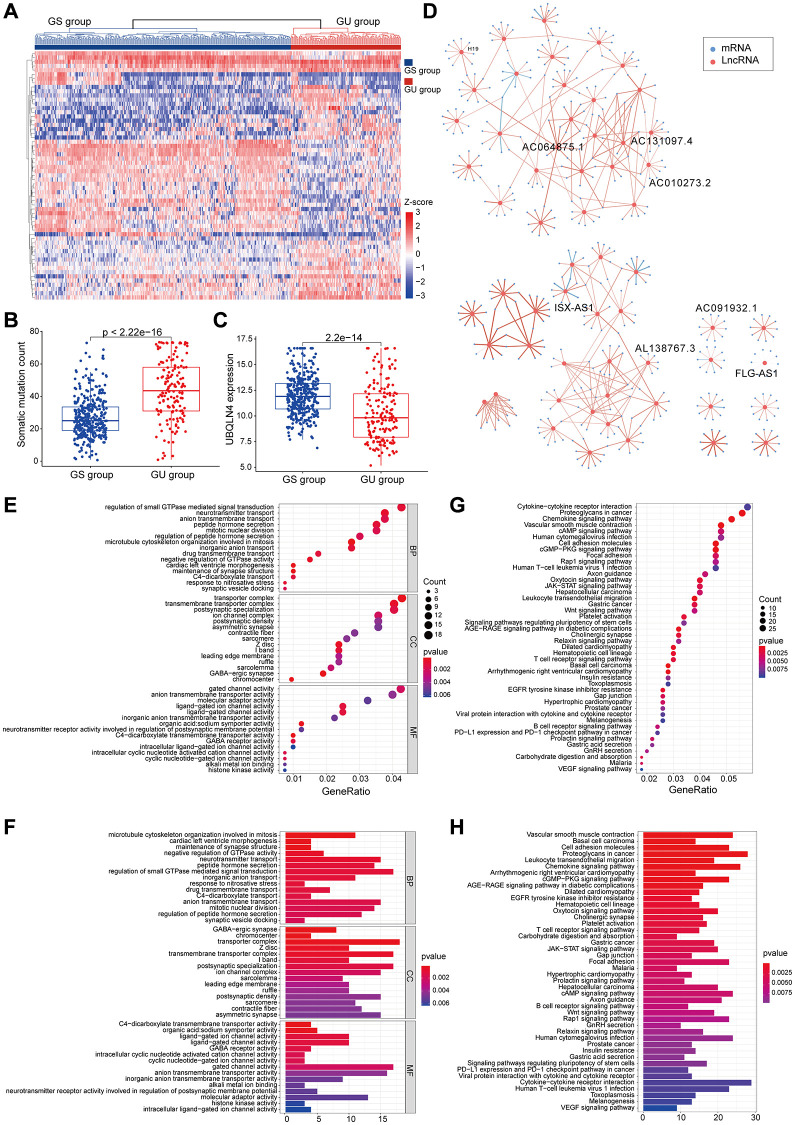
**Identified and functionally interpreted genomic instability-associated lncRNAs in patients with low-grade gliomas.** (**A**) An unsupervised clustering among 529 patients with low-grade glioma was performed based on the expression patterns of 59 candidate genomic instability-associated lncRNAs. The GS group is shown in blue on the left, whereas the GU group is shown in red on the right. (**B**) Box plots for somatic mutations of GS and GU groups. Cumulative somatic mutations in the GU group were significantly higher compared with those in the GS group. (**C**) Box plots of the expression levels of UBQLN4 in the GU and GS groups. Expression levels of UBQLN4 were significantly lower in the GU group compared with the levels in the GS group. (**D**) Pearson correlation coefficient analysis based genomic instability-associated lncRNA and mRNA co-expression network. (**E**–**H**) GO and KEGG functional enrichment analysis of lncRNA co-expression mRNA through.

The flow chart or our work was shown in Figure 1. Frequency of the cumulative number of somatic mutations was calculated for each patient and then sorted in descending order to determine LncRNAs associated with genomic instability. Clinical and pathological characteristics of TCGA-LGG patients were used to explore the clinical outcome of these patients and are presented in Table 1. Patients were divided into (GU) and (GS) groups based on the cumulative number of somatic mutations. lncRNA expression profiles of 137 patients in the GS group and 133 patients in the GU group were compared, and lncRNAs with significantly different expression levels were identified. Analysis using SAM method showed that 59 lncRNAs were differentially expressed (|logFC |>1, FDR adj *p*-value < 0.05, Supplementary Table 1). Unsupervised hierarchical clustering analysis was performed using the 59 differentially expressed lncRNAs for the 529 samples retrieved from TCGA. The 529 samples were divided into two groups based on the levels of the 59 differentially expressed lncRNAs. Analysis showed that the two groups had significantly different somatic mutation patterns (Figure 2A). The GU group showed significantly higher cumulative somatic mutations compared with the GS group. Notably, the GU group showed significantly higher median number of cumulative somatic mutations compared with the GS group (*P* < 0.001) (Figure 2B). In addition, the expression level of UBQLN4 gene [20] (a genomic instability-associated gene overexpressed in aggressive tumors) was compared in the GU and GS groups (Figure 2C).

**Table 1:** Clinical information for three LGG patients sets in this study.

**Covariates**		**Training set (*n* = 240)**	**Testing set (*n* = 237)**	**TCGA set (*n* = 477)**	***P*-value**
Age	<=41 years	129 (53.75%)	117 (49.37%)	246 (51.57%)	0.3864^a^
	>41 years	111 (46.25%)	120 (50.63%)	231 (48.43%)	
Gender	female	113 (47.08%)	103 (43.46%)	216 (45.28%)	0.4821^a^
	male	127 (52.92%)	134 (56.54%)	261 (54.72%)	
First presenting symptom	Headaches	48 (20%)	46 (19.41%)	94 (19.71%)	0.9366^b^
	Mental Status Changes	19 (7.92%)	19 (8.02%)	38 (7.97%)	
	Motor/Movement Changes	19 (7.92%)	16 (6.75%)	35 (7.34%)	
	Sensory Changes	7 (2.92%)	9 (3.8%)	16 (3.35%)	
	Visual Changes	7 (2.92%)	4 (1.69%)	11 (2.31%)	
	Seizures	116 (48.33%)	116 (48.95%)	232 (48.64%)	
	unknown	24 (10%)	27 (11.39%)	51 (10.69%)	
First presenting symptom longest duration	0–30 Days	106 (44.17%)	97 (40.93%)	203 (42.56%)	0.7409^b^
	31–90 Days	33 (13.75%)	39 (16.46%)	72 (15.09%)	
	91–180 Days	19 (7.92%)	15 (6.33%)	34 (7.13%)	
	>181 Days	51 (21.25%)	51 (21.52%)	102 (21.38%)	
	unknown	31 (12.92%)	35 (14.77%)	66 (13.84%)	
Diagnoses	Astrocytoma, anaplastic	58 (24.17%)	63 (26.58%)	121 (25.37%)	0.8172^b^
	Astrocytoma, NOS	29 (12.08%)	29 (12.24%)	58 (12.16%)	
	Mixed glioma	69 (28.75%)	57 (24.05%)	126 (26.42%)	
	Oligodendroglioma, anaplastic	34 (14.17%)	38 (16.03%)	72 (15.09%)	
	Oligodendroglioma, NOS	50 (20.83%)	50 (21.1%)	100 (20.96%)	
Radiation therapy	YES	137 (57.08%)	135 (56.96%)	272 (57.02%)	0.6421^a^
	NO	83 (34.58%)	73 (30.8%)	156 (32.7%)	
	unknown	20 (8.33%)	29 (12.24%)	49 (10.27%)	
Seizure history	YES	142 (59.17%)	140 (59.07%)	282 (59.12%)	0.9998^a^
	NO	83 (34.58%)	82 (34.6%)	165 (34.59%)	
	unknown	15 (6.25%)	15 (6.33%)	30 (6.29%)	
Sample type	Primary Tumor	230 (95.83%)	231 (97.47%)	461 (96.65%)	0.4609^a^
	Recurrent Tumor	10 (4.17%)	6 (2.53%)	16 (3.35%)	
Grade	G2	124 (51.67%)	107 (45.15%)	231 (48.43%)	0.2069^a^
	G3	116 (48.33%)	130 (54.85%)	246 (51.57%)	
IDH1 mutation status	Mutant	42 (17.5%)	48 (20.25%)	90 (18.87%)	0.6728^a^
	Wildtype	18 (7.5%)	16 (6.75%)	34 (7.13%)	
	unknown	18 0 (75%)	173 (73%)	353 (74%)	

^a^Chi square test.

^b^Wilcoxon rank sum test.

The top 10 most relevant mRNAs among the differential lncRNAs in the GS and GU groups were screened out for each lncRNA as its target genes. This dataset of lncRNA-related mRNAs was used to construct a lncRNA-mRNA co-expression network (Figure 2D). GO analysis of LncRNA-related target genes showed that the mRNAs in this network were highly involved in formation and progression of genomic instability, including mitosis, maintenance of synaptic machinery, transporter complexes and activity of various ion channels (Figure 2E and 2F). KEGG pathway analysis of LncRNA-associated target genes identified several signalling pathways associated with genomic instability, tumorigenesis, progression and treatment of low-grade glioma including B cell receptor signalling pathway, T cell receptor signalling pathway, Rap1 signalling pathway, JAK-STAT signalling pathway, PD-L1 expression and PD-1 checkpoint signalling pathway, the Wnt signalling pathway, cAMP signalling pathway and cGMP-PKG signalling pathway (Figure 2G and 2H).

Analysis showed that 59 lncRNAs are implicated in genomic instability. Variations in their expressions may destabilize the cellular genome and disrupt cellular homeostasis of regulatory networks associated with lncRNAs thus inducing various signalling pathways that promote cancer development. The 59 differentially expressed lncRNAs were then referred as genomic instability- associated lncRNAs (GILncRNAs).

### Identification of a genomic instability-mutant lncRNA signature for prognosis using the training set

**Figure 3:**
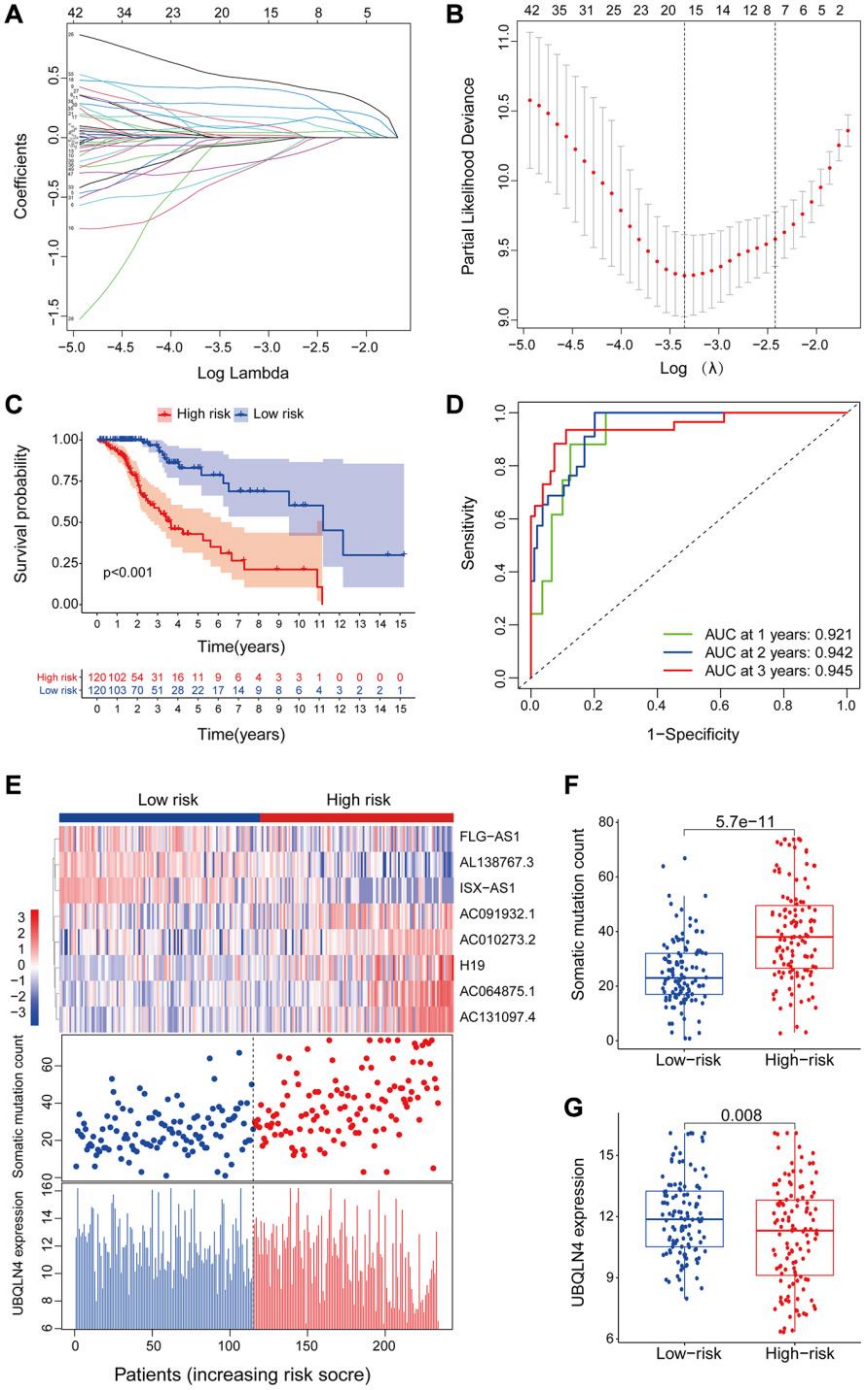
**LncRNAs signature of the genomic instability used to predict outcomes in the training set.** (**A**) Lasso Cox analysis identified 16 lncRNAs associated with genomic instability that were highly associated with prognosis. (**B**) Determination of the optimal value of penalty parameters through 1000 replicates of cross-validation. (**C**) Kaplan-Meier estimation of GILncSig-predicted overall survival of low- or high-risk patients in the training set. (**D**) Time-dependent ROC curves of GILncSig at 1, 2 and 3 years. (**E**) Distribution of cumulative somatic mutations and expression of UBQLN4 in high- and low-risk groups in the GILncSig model of low-grade glioma patients. (**F**) Box plot for distribution of cumulative somatic mutations in the low- and high-risk groups of LGG patients. (**G**) Box plots for UBQLN4 gene expression in low- and high-risk groups of LGG patients.

To further explore the predictive prognosis role of the candidate lncRNAs associated with genomic instability, 477 patients with low-grade glioma from the TCGA project were divided into a training group (*n* = 240) and a testing group (*n* = 237). Expression levels of 59 genomic instability-associated lncRNAs were analyzed using univariate Cox proportional risk regression to identify prognosis associated lncRNAs. Analysis showed 54 genomic instability-associated lncRNAs significantly correlated with prognosis of low-grade gliomas in the training set (*P* < 0.001). Lasso regression analysis was performed using these lncRNAs to avoid over-fitting and 16 lncRNAs associated with genomic instability in LGG were identified (Figure 3A). Notably, the optimal value of the penalty parameter was determined by performing 1000 replicates of cross-validation (Figure 3B). Further, the 16 candidate LncRNAs were screened to identify those with independent prognostic value. A total of eight genomic instability-associated LncRNAs including H19, FLG-AS1, AC091932.1, AC064875.1, AL138767.3, AC010273.2, AC131097.4, and ISX-AS1 were identified as independent prognostic risk factors using stepwise regression multi-factor Cox regression analysis identified (Table 2). A GILncSig was constructed using the coefficients of multifactorial cox analysis and expression levels of the eight independent prognosis-associated LncRNAs for assessing the prognostic risk in low-grade glioma patients. the score was calculated using the formula: GILncSig Risk score = [H19^*^0.0293] + [FLG-AS1^*^–0.5959] + [AC091932.1^*^0.3356] + [AC064875.1^*^0.3690] + [AL138767.3^*^–0.6290] + [AC010273.2^*^0.6908] + [AC131097.4^*^0.7026] + [ ISX-AS1^*^–0.5280].

**Table 2:** Multivariate Cox regression analyses of the 8 of 59 genome instability-related lncRNAs associated with overall survival in LGG.

**Ensembl ID**	**Gene Symbol**	**Genomic location**	**Coefficient**	**HR**	**95% CI**	***P*-value**
ENSG00000130600	H19	chr11:1,995,130–2,001,710	0.029	1.030	1.015–1.045	<0.001
ENSG00000237975	FLG-AS1	chr1:152,168,125–152,445,460	–0.596	0.551	0.276–1.100	0.091
ENSG00000260981	AC091932.1	chr5:8,785,042–8,785,468	0.336	1.399	1.082–1.808	0.010
ENSG00000225649	AC064875.1	chr2:12,780,593–13,007,029	0.369	1.446	1.113–1.879	0.006
ENSG00000225913	AL138767.3	chr10:87,607,985–87,659,383	–0.629	0.533	0.260–1.093	0.086
ENSG00000248664	AC010273.2	chr5:69,113,112–69,159,496	0.691	1.995	1.322–3.012	0.001
ENSG00000235151	AC131097.4	chr2:241,844,380–241,845,036	0.703	2.019	1.258–3.242	0.004
ENSG00000286592	ISX-AS1	chr22:34,756,665–34,997,916	–0.528	0.590	0.399–0.872	0.008

The risk score for an individual invalid in the training set was obtained using GILncSig and LGG patients were then grouped into low- and high-risk groups using the median risk score as the threshold. Kaplan-Meier analysis showed that overall survival of patients in the low-risk group was significantly higher compared with that of patients in the high-risk group. This finding indicates that the prognostic biomarker for predicting risk scores was accurate (*P* < 0.001; Gehan-Breslow-Wilcoxon test: *P* = 4.0207E-9) (Figure 3C). Survival analysis curves showed that the three-year survival rate among LGG patients with high-risk LGG was approximately 56.8% at 95% CI confidence interval of [46.41%–69.6%], and the three-year survival rate for patients in the low-risk LGG group was approximately 94.9% at 95% CI confidence interval of [89.3%–100%]. Furthermore, the five-year survival rate for patients with high-risk LGG was approximately 39.0% at 95% CI confidence interval of [27.2%–55.9%], and the five-year survival rate for patients with low-risk LGG was approximately 78.6%, at 95% CI confidence interval [66.3%–93.3%]. The ten-year survival rate for patients with high-risk LGG was approximately 10.7%, at 95% CI confidence interval [2.25%–50.8%], whereas the low-risk LGG patients had a ten-year survival rate of approximately 60.2% at 95% CI confidence interval of [41.8%–86.6%]. ROC curve analysis of GILncSig over time for 1, 2, and 3 years showed an area under the curve of 0.921, 0.942, and 0.945, respectively (Figure 3D).

Patients in the training set were grouped based on the score and expression level of GILncSig was determined to represent the somatic mutation counts of the patients. A risk heat map, mutation scatters plot and gene expression map were generated to show the relationship between the risk score and gene expression levels of each LGG sample (Figure 3E). The heat map of the expression profiles of the 8 LncRNAs showed that lncRNA FLG-AS1, AL138767.3, and ISX-AS1 were significantly highly expressed in the low-risk group compared with the levels in the high-risk group. On the other hand, lncRNA H19, AC091932.1, AC064875.1, AC010273.2, and AC131097.4 were significantly highly expressed in the high-risk group compared with the low-risk group. Comparative analysis showed significant differences in somatic mutation patterns and UBQLN4 gene expression patterns between invalids in the low- and high-risk groups.

High-risk group patients showed a higher number of somatic mutations compared with the patients in the low-risk group (*P* < 0.001, Figure 3F). Interestingly, UBQLN4 expression level was significantly higher in low-risk patients compared with the level in high-risk patients (*P* = 0.008, Figure 3G).

### Validation of a genomic instability-mutant lncRNA signature for prognosis using the test set and TCGA set

**Figure 4:**
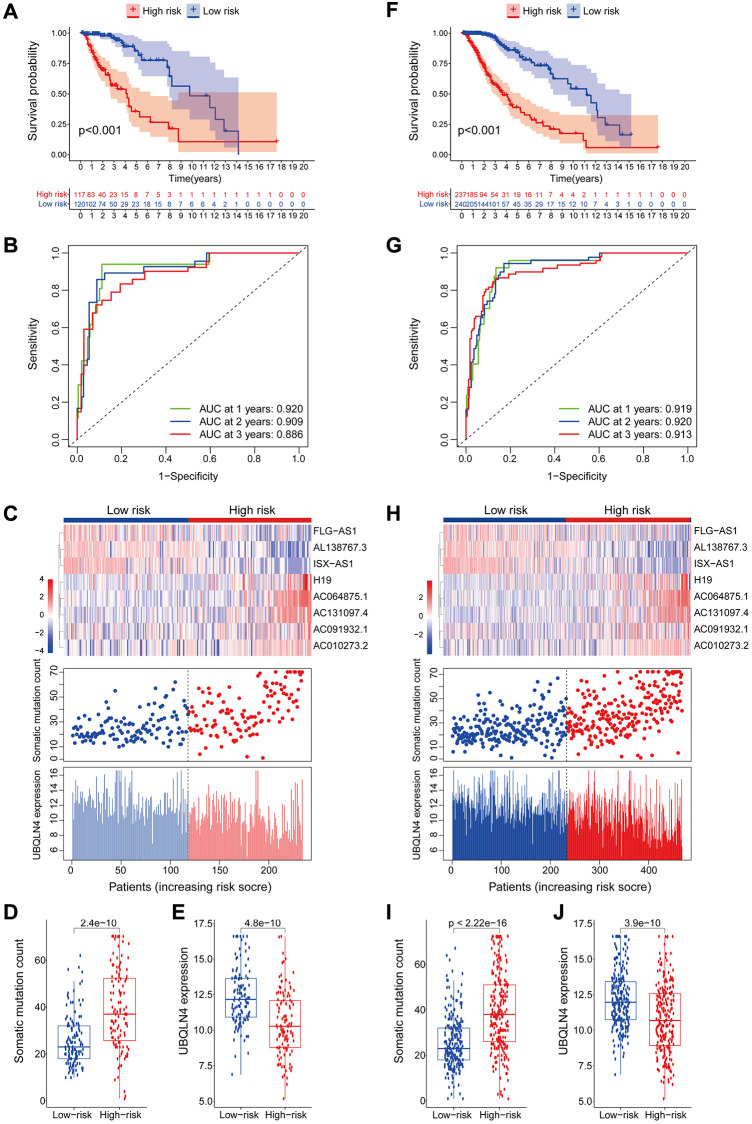
**Validation of the lncRNA signature for genomic instability used to predict outcomes in the testing and TCGA set.** (**A**) Validation of overall survival in low- or high-risk patients predicted by pooling GILncSig with Kaplan-Meier estimates. (**B**) Time-dependent ROC curves of GILncSig at 1, 2 and 3 years in the testing group. (**C**) Verification of LncRNA expression patterns, the profile of somatic mutations and UBQLN4 expression in patients in low- and high-risk groups. (**D**–**E**) Box plots for the distribution of somatic mutations and UBQLN4 expression in high- and low-risk groups of patients. (**F**–**J**) Verification of the above results using the TCGA set.

The GILncSig model was validated using a testing set of 237 patients to test its prognostic performance. Using the same GILncSig and risk thresholds as the training set, the 237 patients in the testing set were grouped into a low-risk group (*n* = 120) and high-risk group (*n* = 117). Kaplan-Meier curves showed that the high-risk group sample had significantly lower OS compared with the low-risk group (*p* < 0.001; Gehan-Breslow-Wilcoxon test: *P* = 2.0904E-9) (Figure 4A). Survival analysis curves showed that the 3- and 5-year survival rates of LGG patients in the high-risk group were approximately 53.8% and 31.1%, at 95% CI confidence intervals of [42.42%–68.3%] and [18.65%–51.8%], respectively. The 3-, 5-, and 10-year survival rates of LGG patients in the low-risk LGG group were approximately 94.1%, 81.7%, and 48.4% at 95% CI confidence intervals of [88.49%–100%], [69.97%–95.4%] and [29.00%–80.6%], respectively. Analysis of the 1-year, 2-year, and 3-year ROC curves for GILncSig in the test group over time showed AUC values of 0.920, 0.909, and 0.886, respectively (Figure 4B). Expression of GILncSig and somatic mutation counts in the test samples are presented in Figure 4C. Significant differences in somatic mutation counts were observed between patients in the low- and high-risk groups (*p* < 0.001, Figure 4D). The high-risk group showed significantly lower expression levels of UBQLN4 compared with the low-risk group (*p* < 0.001, Figure 4E).

GILncSig's prognosis in the TCGA group was similar to the findings of the test group. Patients in the TCGA group were divided into low- and high-risk groups. Overall survival analysis showed that the overall survival time of patients in the high-risk group was significantly less compared with that of the low-risk group (*p* < 0.001, Figure 4F). The survival rates at three, five and ten years for patients with high-risk LGG were approximately 56.65%, 36.74%, and 11.64%, at 95% CI confidence intervals of [48.85%–65.7%], [27.57%–49.0%], and [4.21%–32.2%], respectively. The survival rates at 3, 5, and 10 years for patients with low-risk LGG were approximately 95.5%, at 95% CI confidence intervals of [91.94%–99.2%], [73.98%–91.2%], and [39.74%–73.8%], respectively. The 1-year, 2-year, and 3-year ROC curve analysis of the TCGA group over time showed AUC values at 0.919, 0.920, and 0.913, respectively (Figure 4G). GILncSig expression levels and somatic mutation count in TCGA samples are presented in Figure 4H. Analysis showed a significant difference in somatic mutation pattern between patients in the high- and low-risk groups (*p* < 0.001, Figure 4I). The high-risk group showed significantly lower expression levels of UBQLN4 compared with those for the low-risk group (*p* < 0.001, Figure 4J).

### Construction of 8 genomic instability-mutant lncRNAs signature and correlation with clinical features for Lower-grade glioma

**Figure 5:**
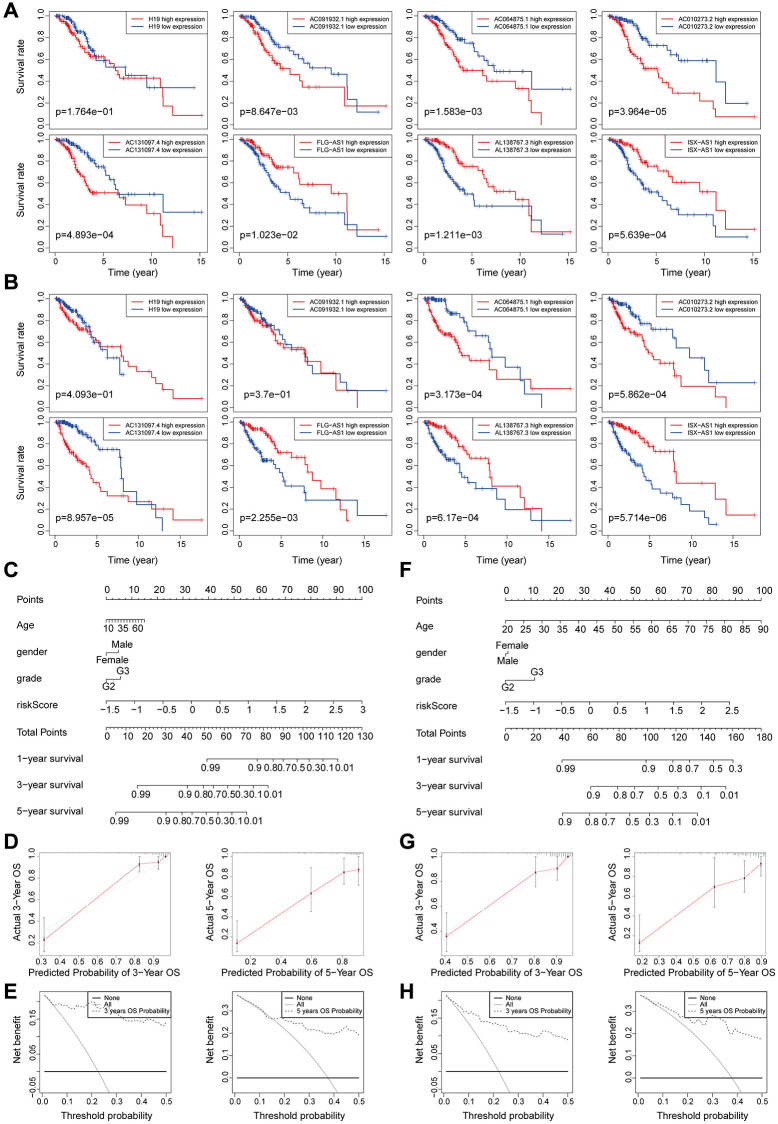
(**A**–**B**) Kaplan-Meier survival curves analysis for the eight genomic instability-associated lncRNAs using the training set (**A**) and validation set (**B**) of patients with low-grade glioma. (**C**–**E**) Nomograms for the eight genomic instability-associated lncRNAs for each factor in the training set, predictions of patient survival at 1, 3, and 5 years. Nomograms were evaluated using calibration curves and DCA curves. (**F**–**H**) Plot nomogram plots in the validation set and evaluation of nomograms using calibration curves and DCA curves.

Correlation between overall survival and the eight genomic instability-associated lncRNA models was determined using Kaplan-Meier curves using the training set (Figure 5A). Analysis showed that lncRNA H19, AC091932.1, AC064875.1, AC010273.2, and AC131097.4 were negatively correlated with overall survival, indicating that they are risk factors for low-grade glioma, therefore, high expression levels of the lncRNAs are associated with poor prognosis. On the contrary, lncRNA FLG-AS1, AL138767.3, and ISX-AS1 were positively correlated with overall survival (*p* < 0.001). These findings show that lncRNA FLG-AS1, AL138767.3, and ISX-AS1 play a protective role in low-grade glioma. Similar findings were obtained through validation using the validation set (Figure 5B). A nomogram was constructed based on tumor grade, gender, age and the eight genomic instability-related lncRNA risk scores in the training group using multivariate Cox regression results (Figure 5C). Analysis of the nomogram, showed that the prognosis-related factors can be used to accurately predict 1-, 3- and 5-year survival of patients. The consistency index (C-index) was used for validation to evaluate the predictive value of the nomogram. Analysis showed that the C-index of the nomogram was 0.8693 and the 95% CI confidence interval of the C-index was [0.8356–0.9030]. The calibration curve (Figure 5D) and clinical decision curve (Figure 5E) showed that the nomogram had a good predictive effect.

Predictive performance of the nomogram was significantly higher compared to that of the risk score model. Similar results were obtained using the validation set (Figure 5F–5H).

### The 8 genomic instability-associated lncRNAs were correlated with infiltration of immune cell subtypes in LGG

**Figure 6:**
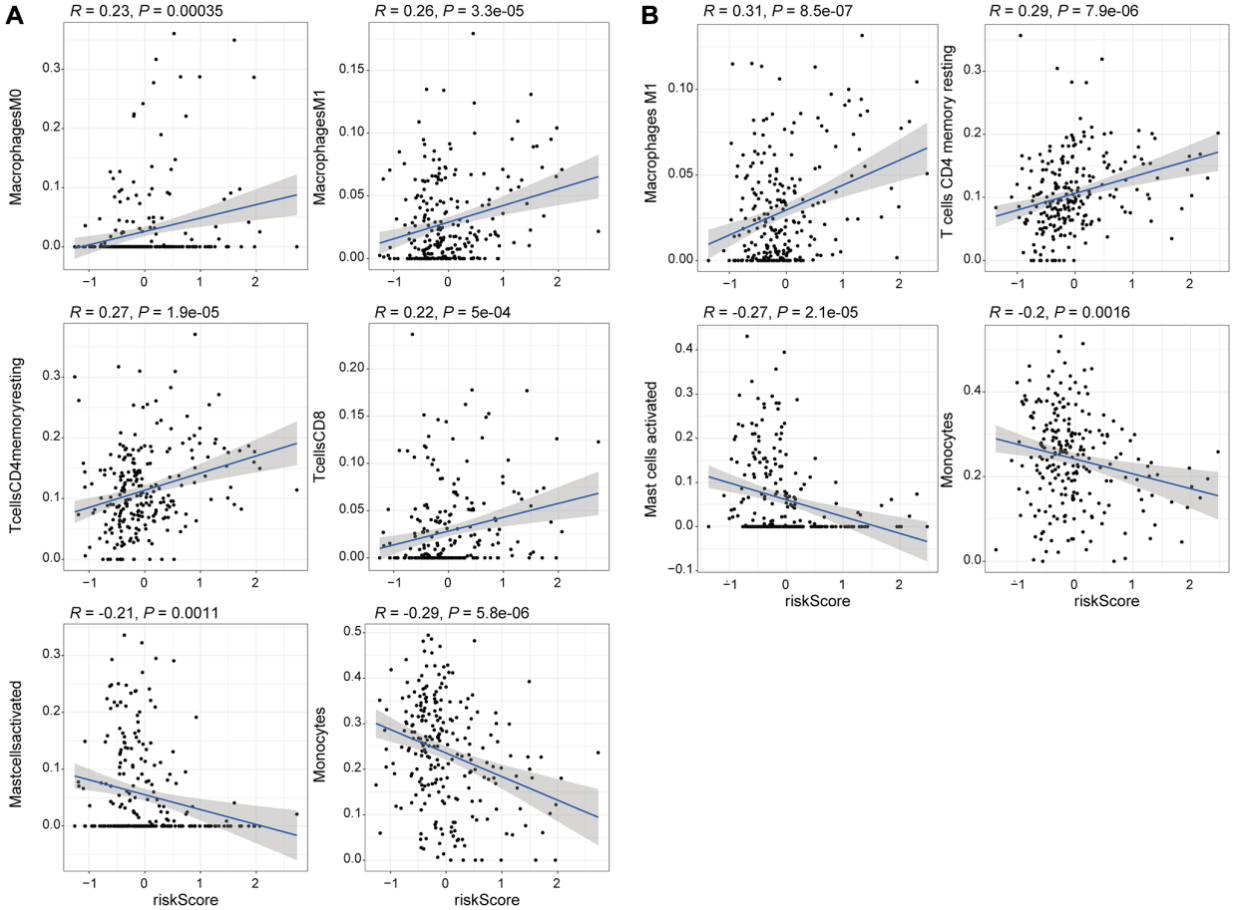
**Correlation analysis of the 8 genomic instability-associated lncRNAs with infiltration of each subtype of immune cells.** (**A**) Correlation coefficients for M0 macrophages, M1 macrophages, memory resting CD4 T cells, and CD8 T cells for the training set were 0.23, 0.26 0.27, 0.22 (*p* < 0.001), whereas the correlation coefficient for activated mast cells was R = –0.21 (*p* < 0.05) and the correlation coefficient for monocytes was R = –0.29 (*p* < 0.001). (**B**) The correlation coefficients among the risk scores for M1 macrophages, memory resting CD4 T cells, activated mast cells and monocytes for the validation set were 0.31, 0.29, –0.27 and –0.2, respectively (*p* < 0.05).

**Figure 7:**
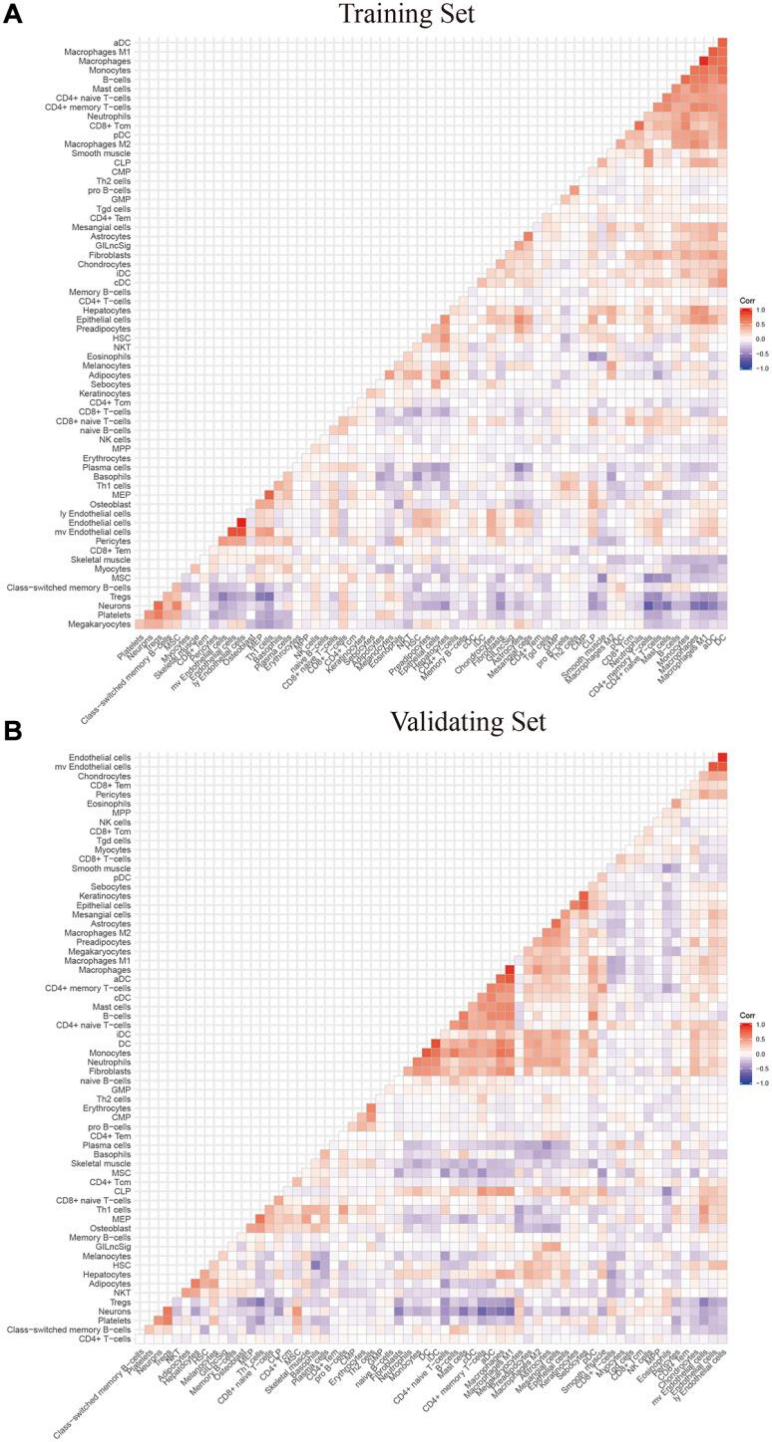
The correlation between GILngSig signature and 64 microenvironment infiltrating immune cells using xcell platform (**A**) training set (**B**) validation set.

To explore the relationship between the expression levels of eight genomic instability-associated lncRNA models and the tumor microenvironment, the abundance of tumor-infiltrating immune subpopulations was determined using the CiberSort algorithm. Furthermore, the correlation between the risk scores of the eight lncRNA-constructed models and infiltration of immune cell subtypes in LGG was analyzed using the training set. The correlation coefficients between macrophages M0, macrophages M1, memory resting CD4 T cells, and CD8 T cells and risk scores were 0.23, 0.26, 0.27, and 0.22, respectively (*P* < 0.001, Figure 6A). These findings indicate that infiltration of tumor immune cells is positively correlated with the risk scores of the eight genomic instability-associated lncRNAs. Activated mast cells (R = –0.21, *p* < 0.05) and monocytes (R = –0.29, *p* < 0.001) were negatively correlated with risk scores of the eight lncRNAs. In addition, verification using the validation set showed that macrophages M1 (R = 0.31, *p* < 0.001) and memory resting CD4 T cells (R = 0.29, *p* < 0.001) were positively correlated with the risk scores of the eight genomic instability-associated lncRNAs. On the other hand, analysis using the validation set showed that activated mast cells (R = –0.27, *p* < 0.001) and monocytes (R = –0.2, *p* < 0.05) immune cell subtypes were negatively correlated with risk scores of the eight genomic instability-associated lncRNAs (Figure 6B). The above correlations between GILncSig signature and the infiltrating immune cells were confirmed with xCell platform in both the training set and validation set (Figure 7A–7B).

### Verification of the genomic instability-mutant lncRNA signature model genes using two external independent LGG datasets

**Figure 8:**
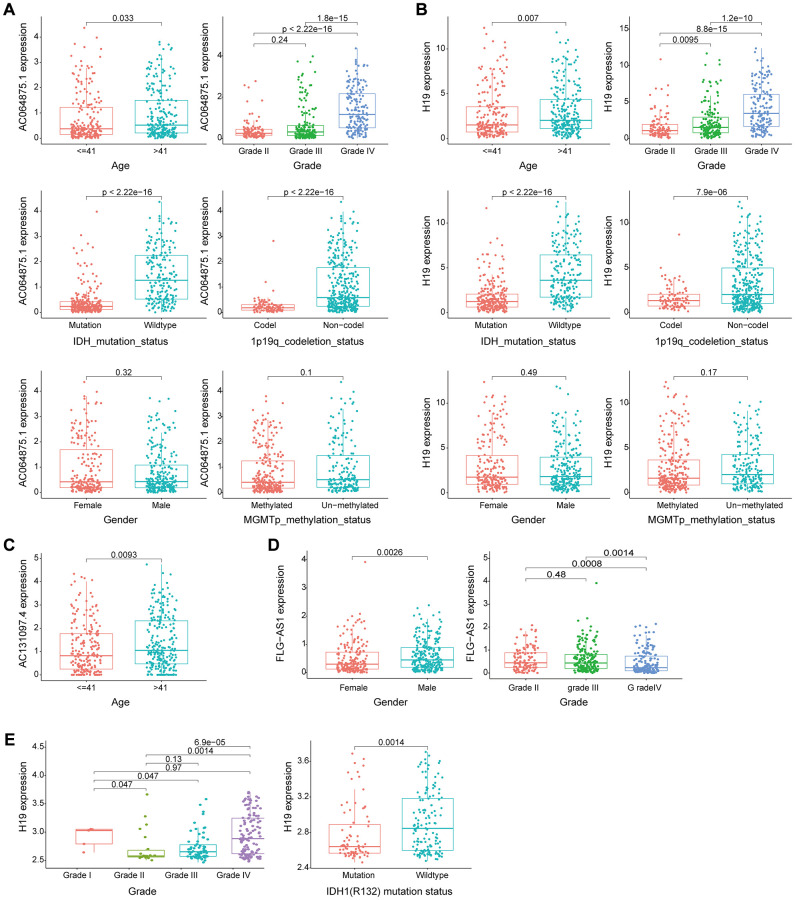
**Evaluation of the performance of the GILncSig partial gene using two external independent CGGA mRNA-seq-693 and GSE16011 datasets.** (**A**–**B**) Box plots for gene expression levels of AC064875.1 and H19 for patients at different ages (< = 41 and > 41 years), tumor grade, IDH1 mutation status, 1p19q chromosome union deletion status, gender and MGMT methylation status in patients from the CGGA mRNA-seq-693 set. (**C**) Box plots for expression of AC131097.4 for patients of different ages in the CGGA mRNA-seq-693 set. (**D**) Box plots for gene expression levels of FLG-AS1 for patients of different tumor grades and gender in the CGGA mRNA-seq-693 set. (**E**). Box plots expression level of lncRNA H19 in patients with different tumor grades and IDH1(R132) mutation status in the GSE16011 dataset.

Cross-platform validation of genomic instability-associated lncRNA models using independent datasets from different platforms showed that only four of the eight lncRNAs in GILncSig (AC064875.1, AC131097.4, FLG-AS1, H19) retrieved from CGGA mRNA-seq-693 represented a large sample size and were matched clinicopathological features. Therefore, we explored the relationship between lncRNA AC064875.1, AC131097.4, FLG-AS1, H19 and low-grade glioma and genomic instability using the CGGA mRNA-seq-693 dataset. Analysis showed that expression levels of AC064875.1 and H19 were significantly correlated with age (< = 41 and >41 years), tumor grade, IDH1 mutation status and chromosome 1p19q joint deletion (*p* < 0.05), however, expression levels of these lncRNAs were not correlated with gender and MGMT methylation status (Figure 8A and 8B). AC131097.4 expression levels were significantly different between age subgroups (*p* < 0.05, Figure 8C). Moreover, FLG-AS1 was significantly correlated with gender and tumor grade (especially between grade II vs IV, III vs IV) (*p* < 0.05, Figure 8D).

Similar findings were obtained using the GSE16011 dataset for lncRNA H19. Expression level of H19 was significantly correlated with tumor grade and IDH1(R132) mutation status (*p* < 0.05, Figure 8E). Survival analysis was performed using lncRNA in the model obtained from CGGA mRNA-seq-693 and GSE16011 datasets to determine their correlation with prognosis. In the CGGA mRNA-seq-693 datasets, the OS of patients with high expression of AC064875.1 and H19 was significantly lower compared with that of patients with low expression levels of AC064875.1 and H19 (*p* < 0.001, Supplementary Figure 1A–1B). On the contrary, high expression level of FLG-AS1 was correlated with a higher overall survival compared with low expression levels of FLG-AS1 (*p* < 0.05, Supplementary Figure 1C). In GSE16011 dataset, patients with low H19 expression level showed significantly higher overall survival compared with the OS of patients with high expression level of H19 (*p* < 0.001, Supplementary Figure 1D). Notably, these findings were consistent with the findings obtained using the TCGA training group and test group.

### Genomic instability-associated lncRNA signature performance compared with existing lncRNA-related signatures in survival prediction

**Figure 9:**
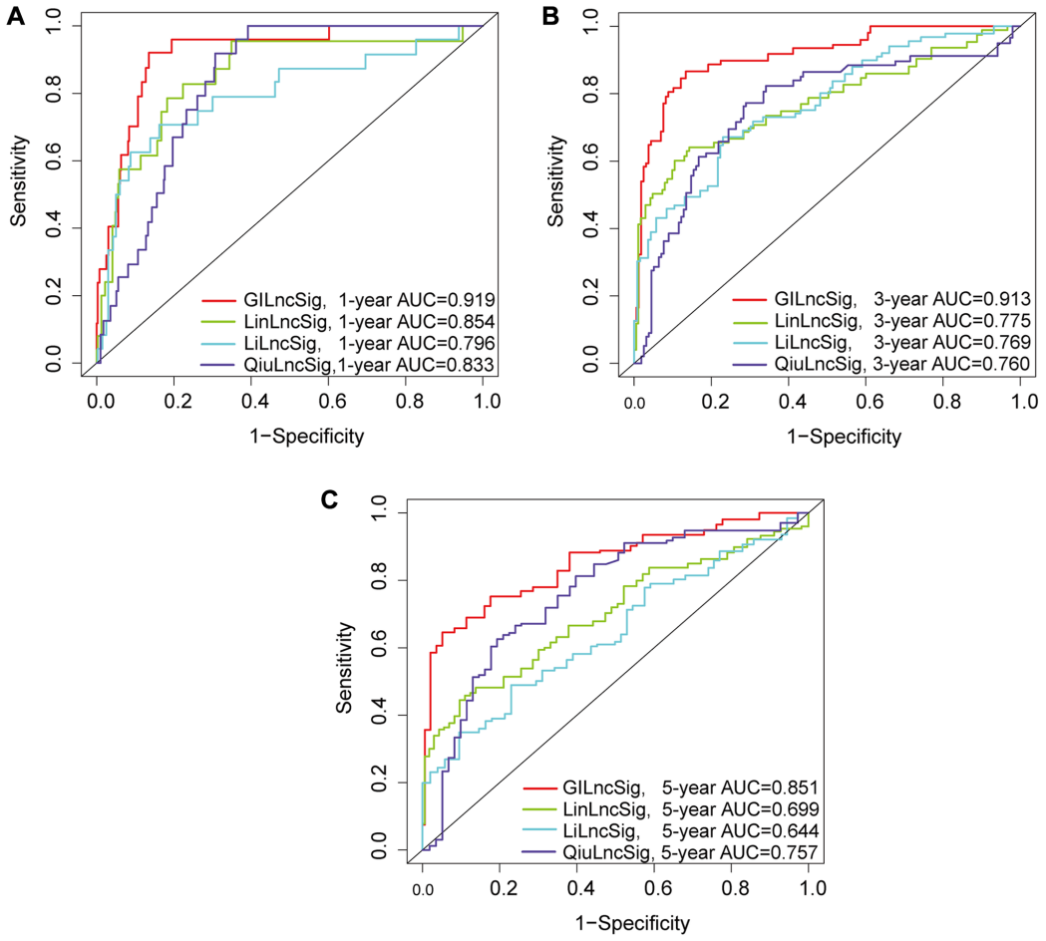
(**A**–**C**) ROC analysis of overall survival at 1-, 2- and 3- years for GILncSig, LilncSig, LilncSig and QiulncSig.

The predictive performance of the genomic instability-associated lncRNA model was compared with three glioma-associated lncRNA signatures reported in previous studies (Table 3). The three signatures included a 6-lncRNA signature reported by Lin's study [21] (hereafter referred to as LinlncSig) and the 8-lncRNA signature from Li's study [22] (hereafter referred to as LilncSig), which used the same cohort of TCGA patients. The third study was a 3-lncRNA signature reported by Qiu's study [23] (hereafter referred to as QiulncSig) which used 167 TCGA-LGG patients with radiological response information. The AUC of GILncSig for one-year OS was 0.919, which was significantly higher compared with the AUC for LinlncSig (AUC = 0.854), LilncSig (AUC = 0.796) and QiulncSig (AUC = 0.833) (Figure 9A). The AUC of GILncSig for three years OS was 0.913 which was significantly higher compared with the AUC for LinlncSig (AUC = 0.775), LilncSig (AUC = 0.769) and QiulncSig (AUC = 0.760) (Figure 9B). Further, the AUC of GILncSig for five years OS was 0.851 which was significantly higher compared with the AUC for LinlncSig (AUC = 0.699), LilncSig (AUC = 0.644) and QiulncSig (AUC = 0.757) (Figure 9C). We turned the GILncSig, LinLncSig, LiLncSig and QiuLncSig signature into the dichotomous variables in order to combine the signatures, which was named as CombinedSig signature. As the ROC curves showed (Supplementary Figure 2A–2C), we found the CombinedSig signature could increase the prognostic power for 1 year and 3 year survival rate compared to other four independent signatures. Although the combined signature could not increase the predictive power for 5 year rate, the AUC values of the GILncSig signature we constructed in this study were still higher than the other three recently published lncRNA prognostic signature as well as CombinedSig signature, indicating that the GILncSig signature had better prognostic performance in predicting survival in LGG patients. We also compared the GILncSig signature with the other clinical factors including Age, Gender, IDH status and Grade. The results showed that whether the prediction of 1 year, 3 year and 5 year, the power of GILncSig signature for predicting prognosis was better than the Age, Gender, IDH status and Grade factors (Supplementary Figure 2D–2F). These findings indicate that GILncSig had significantly higher prognostic performance for survival prediction compared with the three recently published lncRNA markers. The sensitivity and specificity of each GILncSig cut-off value for predicting 5-year survival were shown in Supplementary Table 2.

**Table 3:** Survival predictive value of the GILncSig, LinlncSig, LilncSig and QiulncSig.

	**AUC of 1-year**	**AUC of 3-year**	**AUC of 5-year**
GILncSig	0.919	0.913	0.851
LinlncSig	0.854	0.775	0.699
LilncSig	0.796	0.769	0.644
QiulncSig	0.833	0.760	0.757

Abbreviation: AUC: area under curve.

### Identification of the independence of genomic instability-associated lncRNAs signature in prognostic prediction

**Figure 10:**
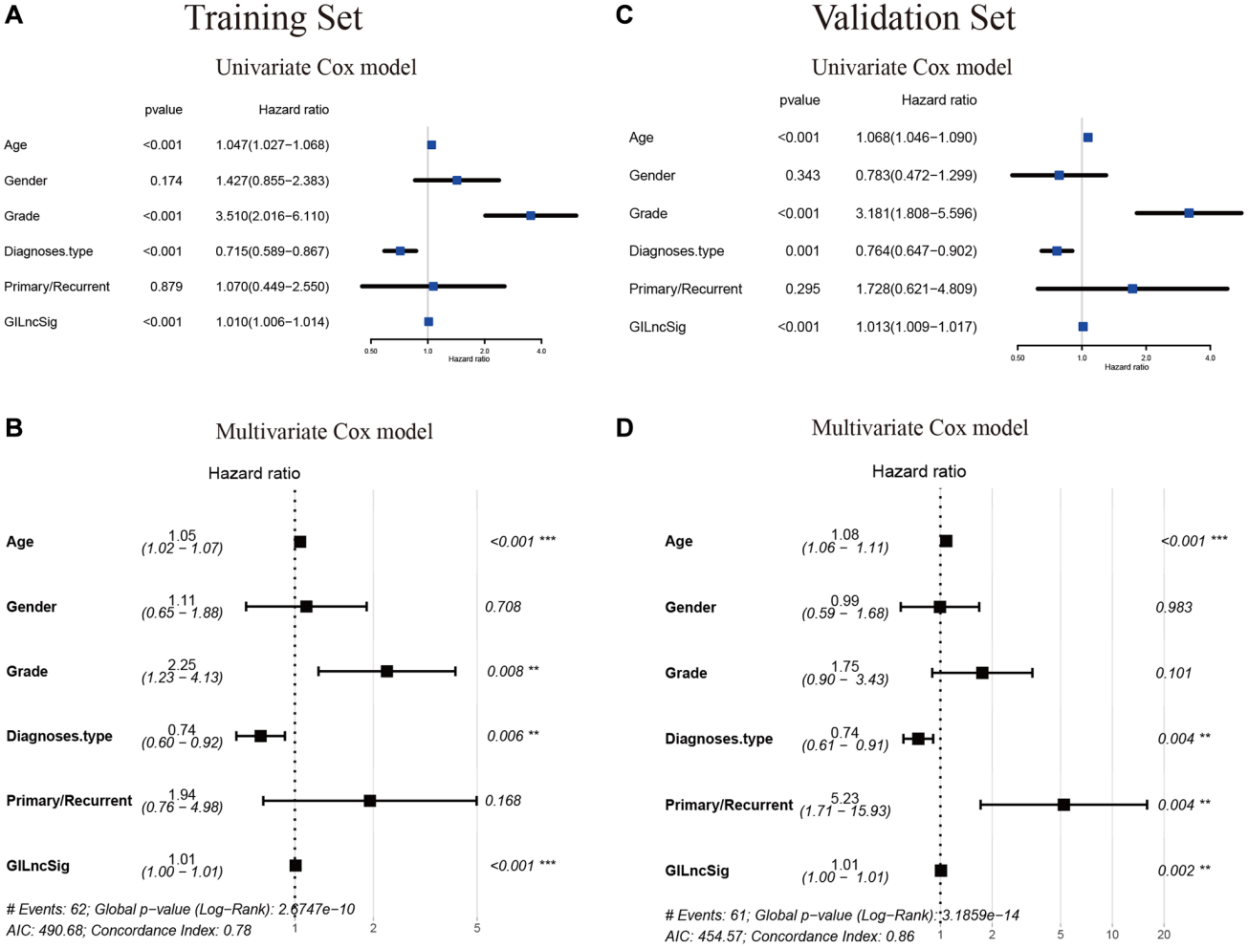
**Verification that the GILncSig is an independent prognostic factor.** (**A**–**C**) The result of univariate Cox regression showed that the age, grade, diagnostic types of gliomas and GILncSig signature were significant prognostic factors for LGG patients. (**B**–**D**) while only the factor of age, diagnostic types and GILncSig signature were also associated with overall survival in the multivariate Cox regression model, indicating that the GILncSig signature was the independent prognostic biomarker for predicting the survival of LGG patients.

A multivariate Cox regression analysis for age, gender, tumor grade, tumor type (primary and recurrent), LGG diagnostic types and GILncSig risk score was performed to see if the GILncSig signature was an independent prognostic predictor. Multivariate analysis showed that GILncSig was significantly correlated with OS in each group in the training set, validation set and TCGA set (*p* < 0.001). In the multivariate analysis, two other clinical factors, namely age and diagnostic types were significant (*p* < 0.05) in addition to GILncSig (Table 4 and Figure 10A–10D).

**Table 4:** Univariate and Multivariate Cox regression analysis of the GILncSig and overall survival in different LGG patient sets.

**Variables**		**Univariable model**			**Multivariable model**		
		**HR**	**95%CI**	***P*-value**	**HR**	**95%CI**	***P*-value**
Training set (*n* = 240)							
GILncSig	High/Low	1.010	1.006–1.014	<0.001	1.01	1.00–1.01	<0.001
Age		1.047	1.027–1.068	<0.001	1.05	1.02–1.07	<0.001
Gender	Female/Male	1.427	0.855–2.383	0.174	1.11	0.65–1.88	0.708
Grade	G2/G3	3.510	2.016–6.110	<0.001	2.25	1.23–4.13	0.008
Diagnoses.type		0.715	0.589–0.867	<0.001	0.74	0.60–0.92	0.006
Primary/Recurrent		1.070	0.449–2.550	0.879	1.94	0.76–4.98	0.168
Testing set (*n* = 237)							
GILncSig	High/Low	1.013	1.009–1.017	<0.001	1.01	1.00–1.01	0.002
Age		1.068	1.046–1.090	<0.001	1.08	1.06–1.11	<0.001
Gender	Female/Male	0.783	0.472–1.299	0.343	0.99	0.59–1.68	0.983
Grade	G2/G3	3.181	1.808–5.596	<0.001	1.75	0.90–3.43	0.101
Diagnoses.type		0.764	0.647–0.902	0.001	0.74	0.61–0.91	0.004
Primary/Recurrent		1.728	0.621–4.809	0.295	5.23	1.71–15.93	0.004
TCGA set (*n* = 477)							
GILncSig	High/Low	1.011	1.008–1.014	<0.001	1.01	1.00–1.01	<0.001
Age		1.057	1.042–1.073	<0.001	1.06	1.04–1.08	<0.001
Gender	Female/Male	1.066	0.746–1.524	0.726	1.08	0.75–1.56	0.679
Grade	G2/G3	3.338	2.254–4.944	<0.001	1.95	1.25–3.02	0.003
Diagnoses.type		0.745	0.658–0.844	<0.001	0.76	0.66–0.87	<0.001
Primary/Recurrent		1.233	0.642–2.368	0.529	2.80	1.42–5.55	0.003

**Figure 11:**
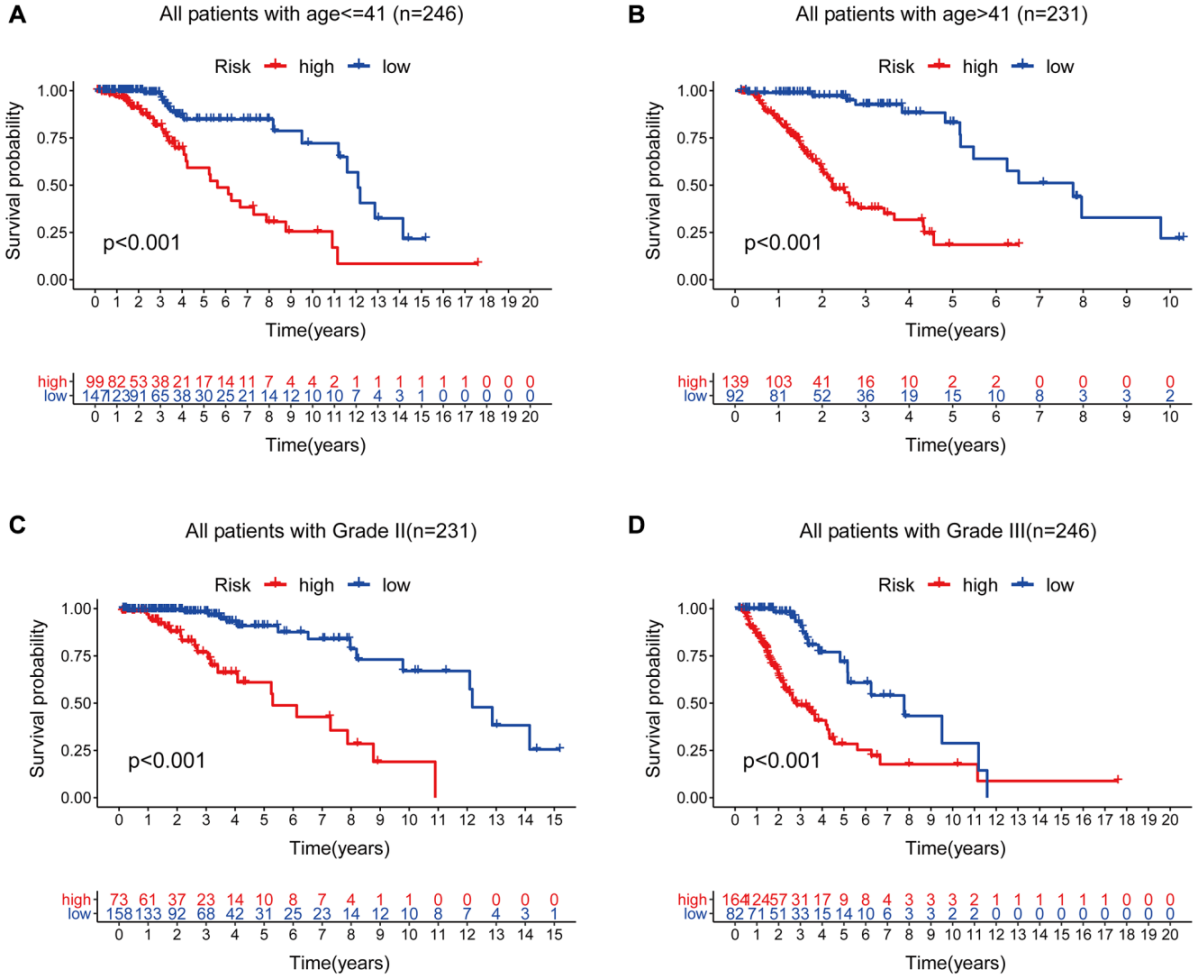
**Stratified analysis by age and tumor grade.** (**A**–**B**) Kaplan-Meier curve analysis of OS in the high- and low-risk groups for patients in the two age groups. (< = 41 and >41 years). (**C**–**D**) Kaplan-Meier curve analysis for OS in high- and low-risk groups for Grade II and Grade III groups.

Therefore, we performed a stratified analysis to determine whether GILncSig prognostic value was independent of age and tumor grade. Patients in the TCGA set were grouped as a young < = 41 years group (*n* = 246) and an age > 41 years group (*n* = 231) based on the median age (41 years). Each age group of patients was further classified as a low- or high-risk group based on GILncSig. The OS of low- and high-risk groups in the young < = 41 years group was significantly different (*p* < 0.001, Figure 11A). In addition, the OS in the age > 41 years group was statistically significant for the low- and high-risk groups (*p* < 0.001) (Figure 11B). All patients with low-grade glioma were then stratified by tumor grade, and patients in the TCGA set were stratified into Grade II (*n* = 231) and Grade III groups (*n* = 246). Patients with pathological grade of Grade II were further grouped into high- and low- groups based on GILncSig score. Analysis showed significant differences in OS between the high- and low- groups (*p* < 0.001, Figure 11C). Furthermore, GILncSig was used to classify pathologically graded Class III patients into high- and low-risk groups, and analysis showed significant difference in OS between the two groups (*p* < 0.001, Figure 11D). These findings indicate that GILncSig is an independent prognostic factor in predicting the overall survival in patients with low-grade gliomas.

### Identification of the relationship between genomic instability-associated lncRNAs signature and IDH1 somatic mutations

**Figure 12:**
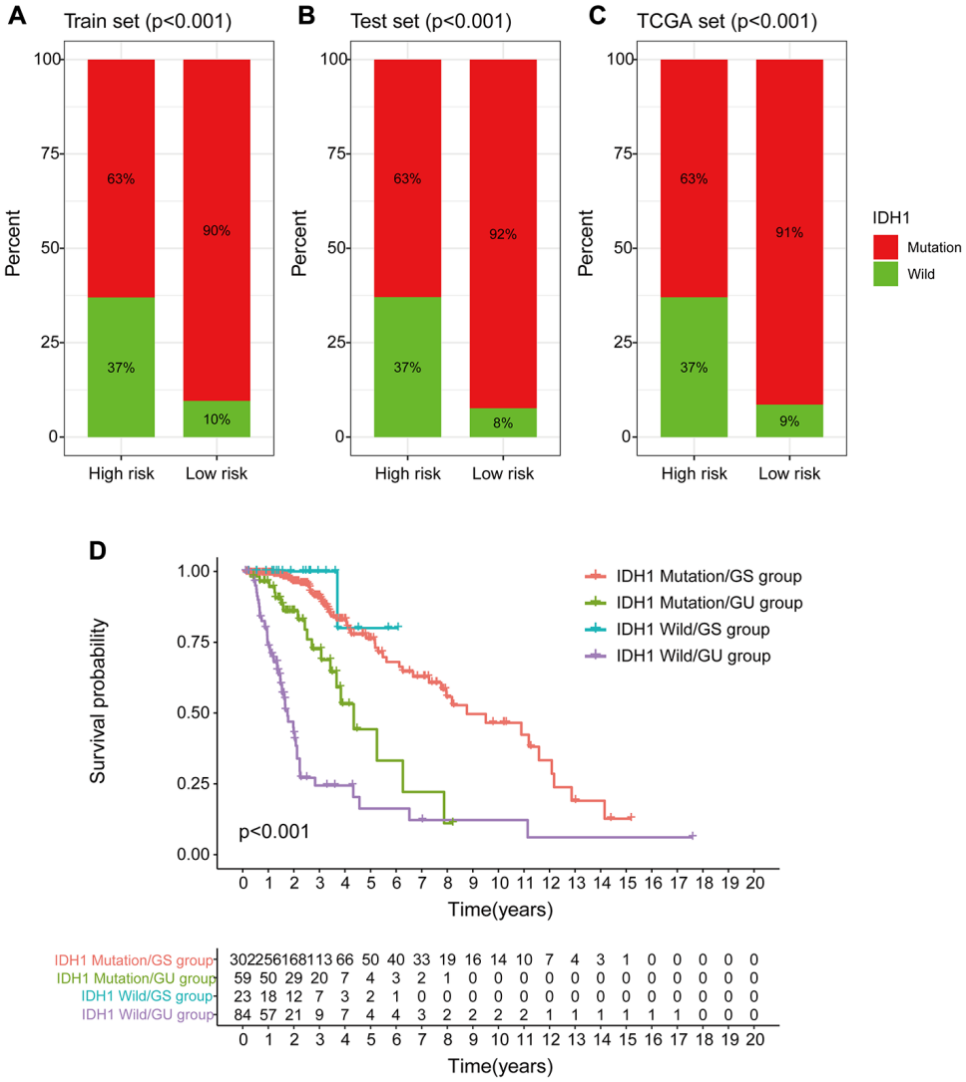
**Correlation between GILncSig and IDH1 somatic mutations.** (**A**–**C**) Proportion of IDH1 mutations in the high- and low-risk groups using the training set, testing set and TCGA set. (**D**) Kaplan-Meier curve analysis of OS of patients with IDH1 mutant status and wild-type status for the combined GS and GU groups.

Chi-square test analysis showed that a significantly higher proportion of patients in the low-risk group possessed IDH1 mutant phenotype compared with the proportion in the high-risk group of the training set, testing set, and TCGA set. In the training set, 90% of patients in the low-risk group had an IDH1 mutation, which was significantly higher compared with the proportion in the high-risk group (63%) (*p* < 0.001, Figure 12A). In the testing set, 92% of patients in the low-risk group had the IDH1 mutation, which was significantly higher compared with the proportion in the high-risk group (63%) (*p* < 0.001, Figure 12B). In the TCGA group, a significantly lower number of patients in the high-risk group (63%) had the IDH1 mutation compared with the number of patients with the IDH1 mutation in the low-risk group (91%) (*p* < 0.001, Figure 12C). These findings indicate that GILncSig is associated with IDH1 mutation status and can be used as a mutational marker for the IDH1 gene. The IDH1 gene mutant is associated with high genomic stability, whereas an increase in the number of wild-type IDH1 genes induces genomic instability, impairs non-homologous end-joining DNA repair, and increases susceptibility to DNA damage [24, 25]. This implies that IDH1 wild type effect on genomic instability can be used to improve the clinical prognosis of low-grade gliomas.

Previous studies report that IDH1 mutations are linked to more prolonged survival and may serve as an independent prognosis biomarker for low-grade gliomas [26, 27]. Therefore, we compared the prognosis performance of GILncSig on patient outcome with prediction of outcome using the IDH1 mutation status. The log-ranch test was used to group the patients into four groups, namely IDH1 Mutation/GS group, IDH1 Mutation/GU group, IDH1 Wild/GS group, and IDH1 Wild/GU group (*p* < 0.001). Use of GILncSig for patients with IDH1 wild-type and IDH mutation showed longer overall survival in the IDH1 Mutation/GU group compared with that for the IDH1 Wild/GU group (Figure 12D). Interestingly, the IDH1 Wild/GS group showed a higher OS rate and had a better outcome compared with that of the IDH1 Mutation/GS group. These findings indicate that GILncSig combined with IDH1 mutation status has greater prognostic significance compared with use of IDH1 mutation status alone.

## DISCUSSION

Low-grade gliomas are complex, heterogeneous intracranial tumors associated with multiple genetic mutations, epigenetic alterations, chromosomal deletions, amplifications, and ectopics. Current personalized treatment for low-grade glioblastoma, comprising surgical treatment combined with radiotherapy with/without temozolomide, increases the two-year survival rate of patients to approximately 27% [28]. Prognostic factors in patients with low-grade gliomas include complex genetic, molecular markers of cancer progression and pathological mechanisms, have been explored as reported in the 2016 World Health Organization (WHO) classification of LGG. These markers group LGG as IDH mutated, 1p/19q co-deficient or 1p/19q preserved, TERT mutated or P53 and ATRX mutated oligodendroglioma cell tumors or astrocytomas; simple TERT mutations and triple-negative tumors, including oligodendroglioma or astrocytoma with no IDH mutation, no co-deletion of 1p/19q and no TERT mutation that is not associated with IDH mutation [29]. IDH mutations are present in nearly 80% of low-grade glioma tumors [30]. IDH mutations are stable markers for LGG progression and prognostic classification, and LGG patients with IDH mutations have significantly longer OS and progression-free survival (PFS) [5]. However, stratification of LGG patients using IDH mutation status is not an effective biomarker for early diagnosis and therapeutic target for LGG. Therefore, there is an urgent need for a robust prognostic model to predict the survival of patients with low-grade gliomas.

Genomic instability drives progression of many tumors [31] and affects prognosis of low-grade gliomas. Genomically unstable tumor cells generate new genetic variants that directly contribute to tumor heterogeneity and resistance to radiotherapy. Poor prognosis of tumors carrying unstable genomes under conventional treatment paradigms implies that the patterns and extent of genomic instability have great prognostic and diagnostic significance for predicting progression and recurrence of tumor. In addition, genomic instability can be used to design novel therapeutic targets. Genomically unstable tumors have a expression level of Neoantigen, a potential target for immunotherapy which can further induce genomic DNA damage in genomically unstable low-grade gliomas, thus it can be used to establish new chemotherapeutic regimens [32].

lncRNAs are widely involved in cancer pathways and several studies are exploring them as potential tumor biomarkers. In addition, lncRNAs are potential therapeutic targets as they are associated with progression of many tumors and prognosis of cancer patients [33]. However, only a few studies have explored the role of genomic instability-associated lncRNAs on prognosis of patients with low-grade gliomas. Therefore, the aim of this study was to model genomic instability-associated lncRNAs for predicting overall patient survival and clinical outcome of LGG patients. In addition, a model containing eight genomic instability-associated lncRNAs (H19, FLG-AS1, AC091932.1, AC064875.1, AL138767.3, AC010273.2 AC131097.4 and ISX-AS1) composed of LncRNA signatures (GILncSig) was constructed. GILncSig grouped patients into low- and high-risk groups with statistically significant differences in overall survival for the training set which was validated using the independent testing set. The GILncSig model predicted significantly better OS in low-risk patients compared with the high-risk patients. Nomogram plots showed that the model was a good predictor of prognosis for 1-, 3- and 5-year OS of low-grade glioma patients. The C-index calibration curve and DCA curve further showed that the model was accurate. The risk-risk model based on eight genomic instability-associated lncRNAs stratified well in LGG was validated by KM curve, ROC curve and risk map analysis. In this study, samples with high-risk scores were significantly correlated with high-risk propensity. In addition, the high-risk group exhibited lower OS compared with the low-risk group, indicating that risk scores are highly correlated with LGG progression and poor prognosis. Further, univariate and multivariate Cox regression analyses showed that the signature was an accurate prognostic factor. The findings of this study showed that lncRNA H19, AC091932.1, AC064875.1, AC010273.2, and AC131097.4 were negatively correlated with overall survival, whereas lncRNA FLG-AS1, AL138767.3, and ISX-AS1 were positively correlated with overall survival (*p* < 0.001), indicating they play a protective role in low-grade gliomas. In addition, genomic instability-associated lncRNA genes were correlated with clinical features of two external datasets.

LncRNA H19 was the first identified RNA regulator implicated in multiple steps of tumorigenesis [34] and is a potential tumorigenic LncRNA for glioma. The expression of H19 non-coding RNA is induced by c-Myc product, a member of the MYC proto-oncogene family, which promotes development of glioma [35]. In addition, H19 acts as a miRNA precursor gene to promote glioma growth, which induces the production of miRNA-675 that further modulates expression of cancer-associated calmodulin 13 (CDH13) [36]. Expression level of H19 is highly correlated with drug resistance in glioma cells. Notably, treatment of gliomas with the drug Temozolomide showed survival of fewer glioma cells and analysis showed low expression levels of H19 [37, 38]. Further studies showed that high expression levels of H19 in glioma cells, promotes development of glioma and invasive metastasis which is consistent with the findings of our study on genomic instability showing a distinct signature. In addition, H19 plays an integral role in evolution of several types of cancer. For example, LncRNA H19 expression is significantly upregulated in primary and metastatic foci of colorectal cancer and is associated with poor prognosis in colorectal cancer. Ectopic H19 expression increases *ex vivo* metastasis of colorectal cancer cells and induces epithelial to mesenchymal transition (EMT) [39]. In addition, lncRNA H19 knockdown inhibits breast cancer cell proliferation and induces apoptosis by regulating miR-130a-3p/SATB1. Furthermore, H19 acts as miRNA-130a-3p, sponge leading to upregulation of SATB1, thus promoting breast cancer progression [40]. Interestingly, H19 interacts with 4E-BP1 at the TOS motif and inhibits 4E-BP1 binding to Raptor competitively, implying that inhibition of pituitary tumors by H19 is more effective compared with carte blanche treatment [41]. AC064875.1 has been used as a new prognostic marker for glioma in recent studies, and high expression level of AC064875.1 was correlated with poor prognosis of patients [42]. Another study reports that lncRNA FLG-AS1 predicts the pathological response and prognosis of neoadjuvant radiotherapy for esophageal squamous cell carcinoma and plays a positive role in prognosis [43]. However, the roles of several other lncRNAs (AC091932.1, AC010273.2, AC131097.4, AL138767.3, ISX-AS1) identified in our GILncSig have not been reported in previous studies. Our validation results on multiple datasets from different databases, findings from previous studies, indicate that GILncSig is a good predictor of the prognosis of cancer patients and serve as an indicator of genomic instability in cancer patients.

Analysis showed that GILncSig significantly distinguishes IDH1 gene wild-type and mutant status and implying that GILncSig can be used to predict the IDH1 mutant status based on the significantly higher IDH1 mutation rate in the low-risk group of patients with genomic instability-associated lncRNAs compared with the high-risk group. In addition, the mutation rate of IDH1 mutant patients was significantly higher compared with that of IDH1 wild-type patients in the low- and high-risk groups (*p* < 0.001). Survival curve analysis after use of GILncSig on IDH1 wild-type and IDH mutant patients separately, showed that prognosis of IDH1 Mutation/GS group > IDH1 Mutation/GU group > IDH1 Wild/GU group (*p* < 0.001). The outcomes showed that prognosis of IDH1 mutation genomic instability group was significantly better compared with that of IDH1 wild genomic instability group. These findings indicate that IDH1 mutation status, combined with genomic instability-associated lncRNA, is a more effective prognostic marker compared with use of IDH1 mutation status alone.

The prognostic value of the eight genomic instability-associated lncRNAs identified in this study has not been fully explored in various cancers. Although our study offers essential insights for determining genomic instability and prognosis in patients with low-grade gliomas, the study had some limitations. Genomic instability plays an essential role in tumor biology, as it increases DSB formation through uncontrolled DNA replication during oncogene activation. Uncontrolled DNA replication results in continued DDR activation allowing cells to virtually enter a state of protective senescence where further proliferation is halted [44]. Oncogene-induced senescence is accompanied by significant alterations in chromatin organization, causing most genome to enter a silent heterochromatin state. Recent studies reported an acutely transformed cDNA from the transcriptome of mouse melanoma cells in clone M3 which was transfected to form foci of tumor-transformed cells, and this RNA was referred as genomic instability-inducing RNA (Ginir). High expression of Ginir in mouse fibroblasts induces genomic instability and oncogenic transformation [45].

Although genomic instability-associated lncRNAs were validated in the TCGA database and the GSE16011 dataset in the CGGA and GEO databases, more independent datasets are needed to validate GILncSig to ensure its reproducibility and robustness. Additionally, roles of these lncRNAs should be further explored through flow cytometry, PCR or IHC. Moreover, to assess the early diagnostic utility of this lncRNA model, the differential expression of the eight lncRNAs in normal and tumor tissues should be evaluated. In addition, further animal studies and cellular experiments should be conducted to test the predictive accuracy of our signature and to explore the mechanisms of genomic instability-associated lncRNAs. Despite these limitations, only a few genomic instability-associated lncRNA signatures have been reported in low-grade gliomas, therefore this signature plays an important role in prediction of survival of LGG patients.

In this study, we constructed a robust and independent prognostic signature based on eight genomic instability-associated lncRNAs. Our results demonstrated that the GILncSig signature was significantly associated with microenvironment infiltrating immune cells and IDH1 mutation status. This study may help improve the power of existing diagnosis and prognosis prediction of low-grade glioma.

## MATERIALS AND METHODS

### Acquisition and processing of low-grade glioma data

Low-grade glioma gene expression profile data, clinical features and somatic mutation information were obtained from TCGA database (https://portal.gdc.cancer.gov), a cancer genome mapping database, and VarScan2, a genomic variant-based investigation platform (GenomeVIP) [46, 47]. VarScan2 is used for analysis of somatic mutations and copy number alterations (CNA) in exome data using tumor-normal tissue pairs. TCGA-LGG cohort comprised 529 low-grade glioma samples, which we identified using the Ensembl database and GENCODE Release 29 (GRCh38.p12) (https://www.gencodegenes.org/human/) annotations were used to discriminate the pattern of low-grade glioma lncRNA copy number alterations and expression [48]. We postulated that most GENCODE lncRNAs (97%) are located in the covered regions. All low-grade glioma samples obtained were randomly divided into training and testing groups. A total of 477 patients were included after excluding patients with incomplete clinical information and survival time less than 30 days. The training set comprised 240 patients, which were used for analysis of lncRNA signatures and generation of a prognostic risk model. A test set of 237 cases was used to independent validation of the performance of the prognostic risk model. Two additional sets for independent verifications of glioma, GSE16011 and CGGA mRNA-seq-693 were acquired from the Gene Expression Omnibus database (https://www.ncbi.nlm.nih.gov/geo/query/acc.cgi?acc=GSE16011) and Chinese Glioma Genome Atlas (http://www.cgga.org.cn/download.jsp), respectively, characterized by large sample sizes and multiple clinicopathological features. All the data we used were quoted from the TCGA and GEO databases, and had obtained the consent of the data submitter. And the patient data in this work were also acquired from the publicly available datasets whose informed consent of patients were complete. All the authors declared that there was no conflict of interest in this study.

### Identification of lncRNAs associated with genome instability in LGG

lncRNA somatic mutation profiles and expression profiles in low-grade glioma genomes were retrieved for identification of mutation-derived binding genomic instability-associated lncRNAs. The proportions of somatic mutations for each LGG individual were calculated then patients were sorted in descending order based on the number of somatic mutation frequencies using Wilcoxon rank-sum test. Further, gene mutation Unsupervised clustering was performed to define the genomic unstable group (GU) which comprised the first 25% of patients and the genomic stable group (GS) was defined as the last 25% of patients. The lncRNAs expression profiles of the GU group and GS group were compared by SAM method (|logFC|>1 and FDR-adjusted with *p* < 0.05) and low-expressed genes were excluded (The mean value of lncRNA expression in all samples was less than 0.5) to determine genomic instability-related lncRNAs.

### Identification of genome instability-associated lncRNAs prognostic signature for low-grade glioma

A univariate Cox proportional risk regression profiling was used to determine the correlation among expression levels of genomic instability-associated lncRNAs and overall survival (*p* < 0.001) of patients with low-grade gliomas. The cox regression analysis was downscaled using the Lasso analysis to further select the best lncRNAs for construction of risk models. A total of 1000 cross-validations were performed to prevent overfitting before identifying the genomic instability-related lncRNAs associated with OS. Multivariate cox regression analysis was then performed to determine the risk coefficients of prognostic markers for lncRNAs associated with genomic instability.

Genomic instability-associated lncRNAs signature (GILncSig) was constructed based on the multiple regression analysis of coefficients and expression levels of lncRNAs associated with genomic instability for prognosis prediction using the following equation:

$$\text{GILncSig Riskscore}=\sum_{i=1}^{n}{\text{coefGILncRNA}}_{i}\times \text{Exp}{\text{ GILncRNA}}_{i}$$

where, GILncSig Risk score represents the prognostic risk score of patients with low-grade glioma. Coef represents multivariate cox regression coefficient, GILncRNA_i_ represents the ith genomic instability-associated lncRNA, whereas Exp GILncRNA_i_ represents the expression level of genomic instability-associated lncRNAs. Further, univariate and multivariate Cox regression analysis were used to determine whether genomic instability-associated lncRNAs are independent predictors for low-grade gliomas.

### Identification and verification of genome instability-associated lncRNAs survival analysis for Low-grade glioma

Patients were grouped into GILncSig low- and high- risk groups using the median scores of LGG patients in training set as risk cutoff values. Kaplan-Meier survival curve analysis was performed to compare differences in overall survival (OS) between the low- and high- groups. Hazard ratios (HRs) and 95% confidence interval (CI) were used to analyze whether the prognoses of the two groups were significantly different. A time-dependent subject receiver operating characteristic curve (ROC) analysis was utilized to compare the specificity and sensitivity of GILncSig risk score for prognosis of patients with low-grade glioma. The above results were then validated using a validation set.

### Construction of a nomogram based on genome instability-associated lncRNAs

Genomic instability-associated lncRNA risk scores and clinical indices were incorporated into the construction of the model to optimize its predictive capability. Nomogram plots were generated to predict patient survival at 1-, 3- and 5- years based on the multivariate Cox regression results. Calibration curves were used to verify its predictive value. The C index and 95% CI confidence interval were evaluated to determine the prediction performance of the nomogram using clinical decision curve analysis (DCA). These results were then validated using the validation set.

### Association of genome instability-associated lncRNAs signature with the immune infiltration

The relationship between lncRNA models and immune cell infiltration was evaluated using expression profiles using ESTIMATE and CIBERSORT algorithms to perform correlation with levels of immune cell subpopulations in the samples [49, 50]. Unsupervised hierarchical clustering of the samples was performed. The infiltrative expression of 22 immune cells in the LGG training set was determined using CiberSort R package. Further, the correlation among immune cell infiltration and risk score was determined using Spearman test. The results were then validated with a validation set to explore the potential mechanisms of different risk groups reflected by GILncSig.

### Functional enrichment analysis

Pearson correlation analysis was performed to establish the correlation between lncRNA and mRNA paired expression, using the top 10 mRNAs that co-expressed lncRNA-associated chaperone proteins. Further, to explore the potential function of lncRNAs, functional enrichment analysis of lncRNA-related mRNA chaperone proteins was performed. The significantly enriched gene ontology (GO) terms and Kyoto Encyclopedia of Genes and Genomes (KEGG) pathways were then identified.

### External validation of genes in the genome instability-associated lncRNAs signature model

Validation of the signature model genes was performed using CGGA mRNAseq-693 tool in the CGGA database and the GSE16011 dataset from GEO database. The equation used to calculate risk scores was used for validation. Kaplan-Meier curve analysis was performed, and expression of genes of different ages, genders, grades and IDH1 mutation status were presented in boxplots.

### Statistical analysis

All statistical analyzes and plots were performed or generated using the R software version 4.0.3. We used the Pearson correlation analysis to determine the relationship between the two. "Limma" R package was used for differential gene screening. The "glmnet" package was used for LASSO Cox regression model analysis. The "survivor" and "survminer" packages were used for perform survival data analysis and for generation of Kaplan-Meier plots. A two-tailed log-rank test was used to determine statistical significance of survival curves. ROC analysis was performed using the "TimeROC" package. The "rms" package was used to construct the Nomogram and for generation of calibration curves. The "clusterProfler" package was used for GO and KEGG enrichment analysis. *P* < 0.05 was statistically significant. We used non-parametric tests to deal with non-normal distributions, and used parametric tests to deal with normal distributions.

## Supplementary Materials

Supplementary Figures

Supplementary Table 1

Supplementary Table 2
