# Supplementary material for: Identification and validation of a novel eight mutant-derived long non-coding RNAs signature as a prognostic biomarker for genome instability in low-grade glioma
Source: Aging (Albany NY). 2021 Jun 3;13(11):15164–92. doi: 10.18632/aging.203079 (PMC8221298; doi:10.18632/aging.203079)
Supplement: Supplementary Figures [file aging-13-203079-s001.pdf]

SUPPLEMENTARY FIGURES

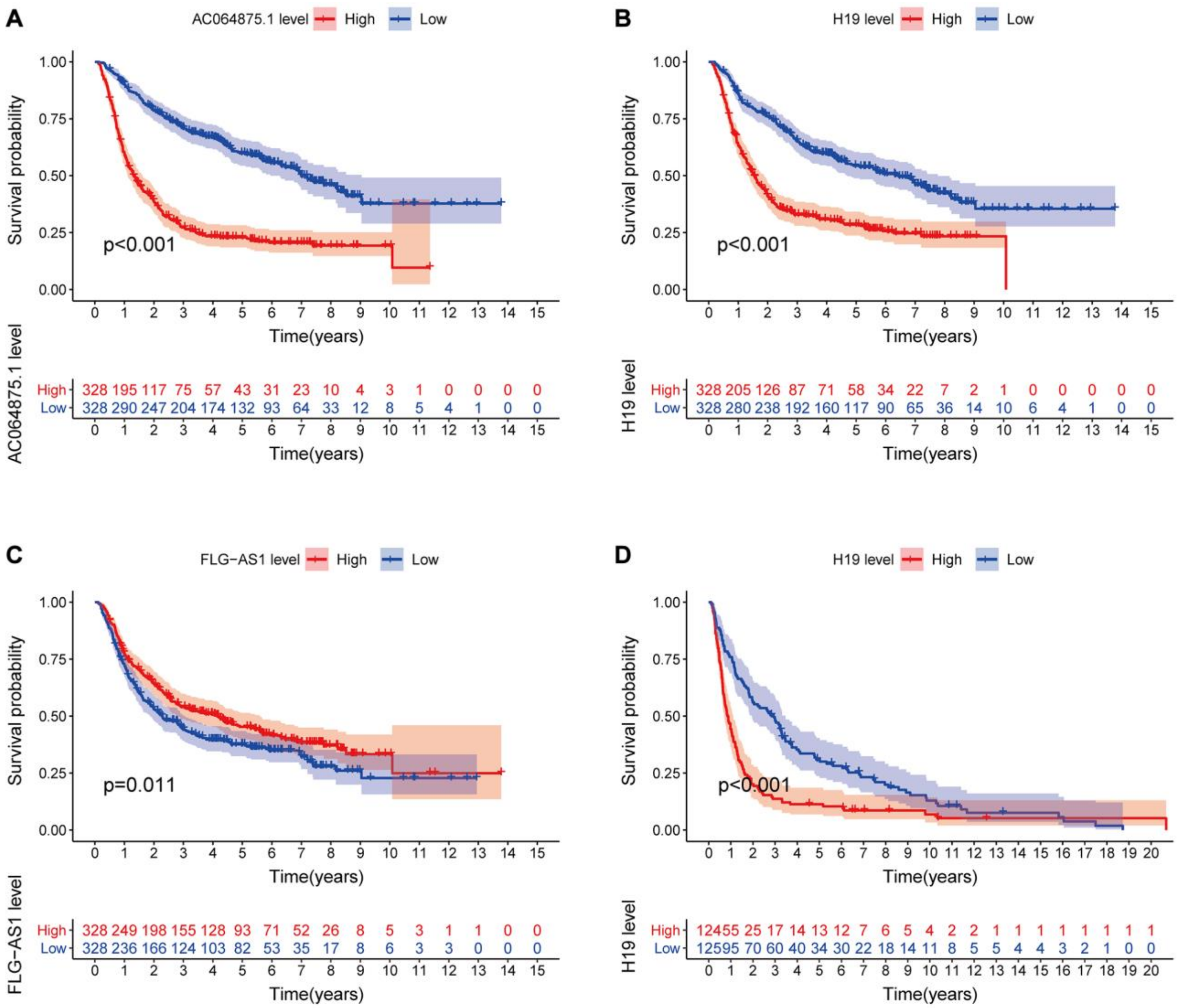

Supplementary Figure 1. (**A**–**B**) Overall survival probability of patients with high expression levels of AC064875.1 and H19 in the CGGA mRNA-seq-693 dataset was significantly lower compared with that of patients with low expression levels (p < 0.001). (**C**) Overall survival probability of patients with high expression level of FLG-AS1 in the CGGA mRNA-seq-693 dataset was significantly higher compared with the low expression level of FLG-AS1. (P < 0.05). (**D**) The overall survival probability of patients with low expression level of H19 in the GSE16011 dataset was significantly higher compared with the OS of patients with high expression of H19. (p < 0.001).

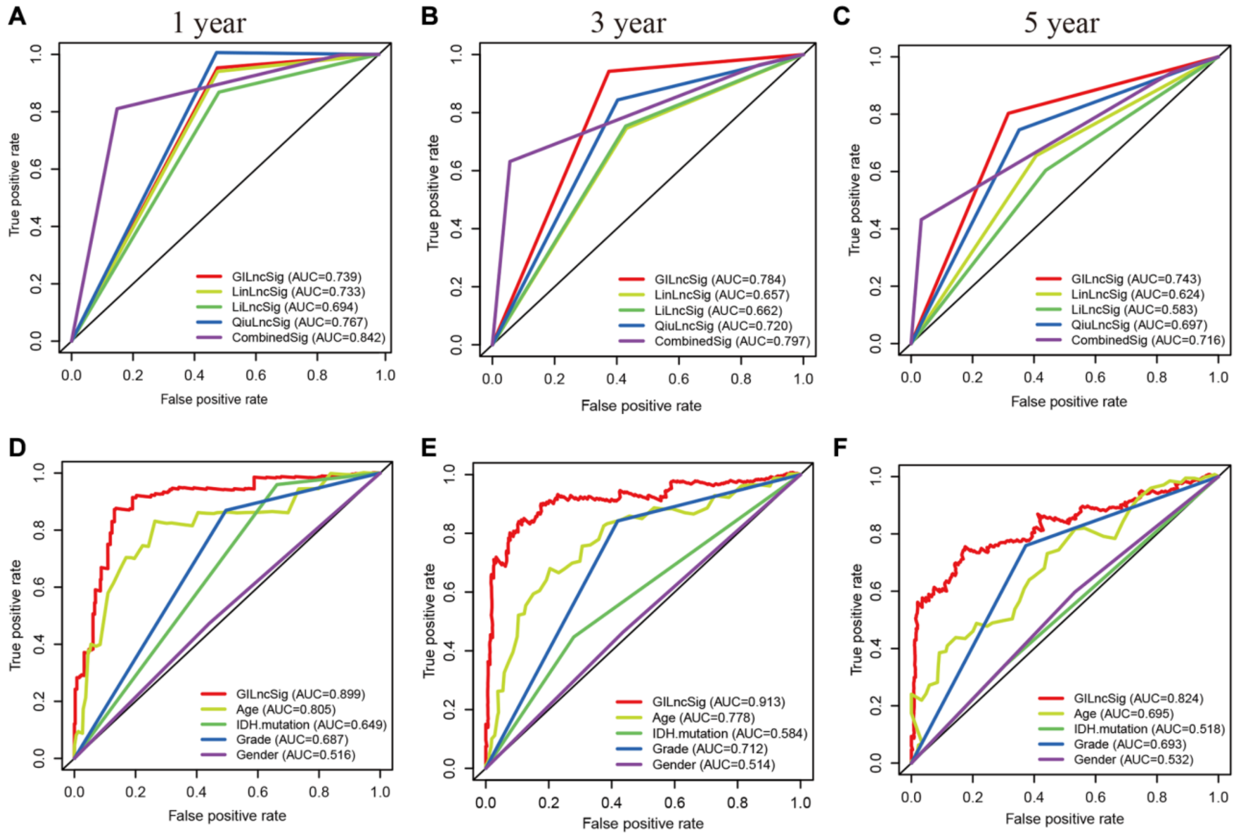

Supplementary Figure 2. (**A**–**C**) As the ROC curves showed, the CombinedSig signature could increase the prognostic power for 1 year and 3 year survival rate compared to other four independent signatures. (**D**–**F**) Receiver operating characteristic (ROC) curve analysis shows that the prediction of 1 year, 3 year and 5 year in the prognostic accuracy of clinicopathological parameters such as age, gender, IDH status and grade and GILncSig signature prognostic risk score.
