# Supplementary material for: Identification and validation of a novel eight mutant-derived long non-coding RNAs signature as a prognostic biomarker for genome instability in low-grade glioma
Source: Aging (Albany NY). 2021 Jun 3;13(11):15164–92. doi: 10.18632/aging.203079 (PMC8221298; doi:10.18632/aging.203079)
Supplement: Supplementary Table 1 [file aging-13-203079-s002.pdf]

SUPPLEMENTARY TABLE

Supplementary Table 1. The differentially expressed genomic unstable and genomic stable related lncRNAs in lower-grade glioma patients.

lncRNA	conMean	treatMean	logFC	*p* Value	FDR
LINC01007	0.740307871	0.168018	–2.139513597	0.0009411	0.00340202
AC109439.2	2.395596545	0.644303	–1.894574393	7.50E-20	1.00E-16
AC002428.2	1.333921383	0.431929	–1.62680791	1.02E-15	2.48E-13
LINC02217	0.66787853	0.240895	–1.471180853	0.0005697	0.002244542
DNMBP-AS1	1.066865411	0.401631	–1.40943686	8.01E-16	2.48E-13
LINC02058	1.58744	0.617127	–1.363063185	3.27E-16	1.25E-13
AL354863.1	1.089323271	0.432545	–1.332510567	7.06E-07	7.61E-06
ISX-AS1	1.021477208	0.414219	–1.302192683	1.43E-09	4.14E-08
AC023421.1	1.608994161	0.668245	–1.267711123	5.76E-06	4.52E-05
AL138767.3	0.814245354	0.340586	–1.257443277	3.59E-14	5.64E-12
ANKRD62P1-PARP4P3	0.776817632	0.330067	–1.234818558	2.04E-09	5.15E-08
AL391845.2	1.148031042	0.488924	–1.231479834	4.52E-14	6.71E-12
AC125616.1	1.074019887	0.457844	–1.23009128	6.29E-08	9.84E-07
AL031710.1	0.809037217	0.347418	–1.219532567	5.44E-07	6.22E-06
AC097641.1	0.577981177	0.249994	–1.209126788	4.43E-12	3.01E-10
AC104024.2	0.780439243	0.338927	–1.203313713	0.00698	0.017782305
AC087442.1	1.129076825	0.516902	–1.127179583	1.52E-15	3.39E-13
AL139246.1	0.776730928	0.358061	–1.117209634	6.76E-11	2.82E-09
AC061961.1	1.246362108	0.5815	–1.099872909	5.22E-07	6.02E-06
AC018410.1	0.717255386	0.337371	–1.088150211	2.08E-08	3.83E-07
FLG-AS1	0.566102043	0.266784	–1.08539195	9.14E-13	7.88E-11
AC009118.1	0.8900668	0.42445	–1.068319556	2.72E-09	6.55E-08
AC124854.1	1.705458064	0.820497	–1.055589518	6.39E-18	5.69E-15
AL390786.1	0.848419029	0.409249	–1.051798604	3.34E-12	2.41E-10
LINC00836	3.672622495	1.797571	–1.030761536	1.68E-12	1.32E-10
RNF219-AS1	1.830454824	0.897093	–1.028873419	1.02E-15	2.48E-13
AL139246.4	0.707122635	0.349126	–1.018210715	1.37E-08	2.71E-07
AL121956.4	0.937556362	0.463795	–1.015419647	8.14E-11	3.35E-09
AL121821.1	0.985791003	0.487908	–1.01467392	1.30E-10	4.83E-09
PIK3CD-AS2	0.269837475	0.539894	1.000585509	4.36E-08	7.33E-07
AC110015.1	0.268027521	0.54466	1.022973919	1.12E-06	1.13E-05
AC091057.1	0.291361296	0.593392	1.026175261	2.57E-14	4.29E-12
AC010273.2	0.468871731	0.959781	1.033511818	3.97E-10	1.35E-08
MIR4435-2HG	0.51666879	1.081763	1.066072864	8.51E-11	3.45E-09
AC016168.2	0.261125127	0.558021	1.095578471	2.93E-07	3.61E-06
AL355974.2	3.247179752	7.105989	1.1298482	1.46E-09	4.16E-08
AP000696.1	0.338403992	0.750695	1.149481128	5.88E-08	9.47E-07
SLCO4A1-AS1	0.318637508	0.718064	1.172197009	0.0131875	0.030202932

AL355974.3	1.528651135	3.485717	1.189196285	2.89E-08	5.08E-07
LINC02308	0.529617356	1.305029	1.301059411	1.21E-10	4.55E-09
AC091932.1	0.28513877	0.740863	1.377541821	2.42E-06	2.19E-05
AJ011932.1	0.332752511	0.872696	1.391029184	5.75E-10	1.83E-08
AL512785.1	0.339901779	0.930143	1.452334171	3.45E-05	0.000207188
CRNDE	0.858686284	2.468972	1.523707283	6.96E-15	1.33E-12
FOXD3-AS1	0.690153292	2.027998	1.555067621	5.27E-11	2.35E-09
AC131097.4	0.22318894	0.693529	1.635691708	0.0010957	0.003836379
AC025171.5	0.269306355	0.850237	1.658615922	5.13E-09	1.13E-07
HOXD-AS2	0.298261437	0.954926	1.678811181	7.01E-17	3.75E-14
AL035446.1	0.707730748	2.295653	1.697631989	6.18E-08	9.79E-07
LINC01579	0.401522107	1.311422	1.707580169	1.01E-05	7.28E-05
AGAP2-AS1	1.111059598	3.814722	1.779641793	0.0001462	0.000718734
AL049871.1	0.167635677	0.676832	2.01346857	5.88E-09	1.28E-07
AC002454.1	0.160563184	0.654202	2.026594269	0.0014202	0.004713457
HOTAIRM1	0.59097175	2.463736	2.059686487	3.79E-08	6.49E-07
AC064875.1	0.269906847	1.127583	2.062700604	9.62E-08	1.41E-06
LINC01831	0.154674658	0.734951	2.248411301	2.08E-13	2.42E-11
LINC01956	0.164126221	0.827916	2.334678097	2.35E-21	6.29E-18
LINC02587	0.315083274	2.130638	2.757480259	0.0057771	0.015225469
H19	0.285984353	2.744758	3.262670572	0.0003098	0.001367447
